# Integrative multi-omics analysis of IFNγ-induced macrophages and atherosclerotic plaques reveals macrophage-dependent STAT1-driven transcription in atherosclerosis

**DOI:** 10.3389/fimmu.2025.1590953

**Published:** 2025-06-18

**Authors:** Mahdi Eskandarian Boroujeni, Natalia Lopacinska, Aleksandra Antonczyk, Katarzyna Kluzek, Joanna Wesoly, Hans A. R. Bluyssen

**Affiliations:** ^1^ Human Molecular Genetics Research Unit, Institute of Molecular Biology and Biotechnology, Faculty of Biology, Adam Mickiewicz University, Poznań, Poland; ^2^ Laboratory of High-Throughput Technologies, Faculty of Biology, Adam Mickiewicz University, Poznań, Poland

**Keywords:** atherosclerosis, IFNγ signaling, STAT1, multi-omics integration, macrophages, single cell RNA-seq, diagnostic markers, gene signature

## Abstract

Atherosclerosis is a chronic inflammatory disease of blood vessels, characterized by atherosclerotic lesions in large- and medium-sized arteries. IFNγ is a crucial mediator of atherosclerosis through activation of signal transducer and activator of transcription (STAT)1. Macrophages (MØ), in different subtypes, play a central role in atherosclerosis, from early foam cell formation to advanced plaque development and potential rupture. Recent evidence in MØ supports a collaborative role of STAT1 with PU.1, in association with histone acetylation and methylation marks, in MØ-specific IFNγ-activated transcriptional responses. This study investigated the role of MØ STAT1-mediated signaling in atherosclerosis progression through multi-omics integration of IFNγ-induced MØ and expression analysis in human and mouse atherosclerotic lesions. First, by integrating ATAC-seq, ChIP-seq, and RNA-seq data from IFNγ-treated and untreated bone marrow-derived MØ, we identified 1139 STAT1-dependent integrative genes. Active transcription of these genes was characterized by prominent promoter STAT1-PU.1 co-binding, increased histone methylation and acetylation and chromatin accessibility. Moreover, KEGG-analysis unraveled a strong connection to lipid metabolism and atherosclerosis-related pathways, whereas STARNET analysis identified high association with LDL cholesterol and diseased vessel traits. Using scRNA-seq data analysis of human carotid and coronary atherosclerotic lesions revealed dynamic changes of STAT1-dependent integrated genes in MØ subtypes, including foamy MØ, monocytes, inflammatory MØ, tissue resident MØ and conventional dendritic cells. Comparative MØ-dependent expression analysis in aortic lesions from LDLr-/- and ApoE-/- high fat diet mouse models substantiated overlap between human and mouse atherosclerosis and identified 24 MØ-specific commonly expressed STAT1-dependent integrated genes. Collectively, we provide detailed insights into MØ-specific IFNγ-activated transcriptional changes, mediated by STAT1-PU.1 co-binding and associated epigenetic changes, and offer the identification of new biomarkers and therapeutic targets in atherosclerosis. Moreover, we present a novel STAT1-dependent gene signature that could potentially serve to monitor MØ-dependent plaque progression during human atherosclerotic disease.

## Introduction

Atherosclerosis is a chronic inflammatory disease of blood vessels, characterized by atherosclerotic lesions in large- and medium-sized arteries, including aorta, and coronary and carotid arteries. Previous studies have shown that atherosclerosis is a lipid-driven chronic inflammatory disease, which involves complex interactions between various immune and vascular cell types and signaling pathways. Among these, the role of macrophages (MØ) and interferon-gamma (IFNγ) signaling has emerged as a critical factor in atherosclerotic lesion formation and progression (1–4).

Recent advances in multi-omics technologies have provided unprecedented opportunities to investigate the molecular mechanisms underlying atherosclerosis at various levels of biological organization. By integrating data from chromatin accessibility assays, epigenetic modifications, transcription factor binding patterns, and gene expression profiles, researchers can now gain a more comprehensive understanding of the regulatory networks driving disease progression (5–8).

One key player in the IFNγ signaling pathway is the transcription factor Signal Transducer and Activator of Transcription 1 (STAT1). STAT1 homodimers, known as γ-activated factor (GAF), directly activate transcription of target genes containing the IFNγ-activated sequence (GAS; consensus TTTCNNNGAAA). STAT1-STAT2 heterodimers together with interferon regulatory factor (IRF)9 (known as ISGF3) expands the range of regulatory elements that can be targeted by STAT1 to the IFN-stimulated response element (ISRE; consensus AGT TTC N2TTTCN). Also, IRF1, as a STAT1-target gene, has been shown to regulate transcription of genes in response to IFNγ. Thus, IRF1 participates in secondary IFNγ responses by activating transcription of ISRE-containing genes. Together, these different STAT1-dependent complexes mediate transcriptional regulation of genes involved in inflammation, lipid metabolism, and immune responses (4, 9, 10). However, the precise mechanisms by which STAT1-mediated IFNγ signaling contributes to atherosclerosis progression remain incompletely understood.

IFNγ is present in atherosclerotic lesions, primarily produced by T cells, natural killer cells and MØ, and has emerged as an important factor in atherogenesis. For example, in the atherosclerotic plaque local environment, IFNγ activates multiple cells of the innate and adaptive immune response, which triggers production of a cascade of pro-inflammatory molecules, including multiple interleukins, chemokines and adhesion molecules. This facilitates recruitment of monocytes to the endothelial wall where they can breach the activated EC monolayer and differentiate into MØ. IFNγ subsequently promotes MØ polarization to the classically activated M1 pro-inflammatory phenotype, which predominates in atherosclerotic lesions, and is considered to be a significant contributor to lesion progression. At the same time, IFNγ also stimulates expression of scavenger receptors and suppression of reverse cholesterol transport proteins in MØ, thereby promoting abnormal accumulation of modified LDL (mLDL) and suppression of cholesterol efflux to HDL, which leads to foam cell formation. Subsequent IFNγ-driven foam cell apoptosis, causing lipid overload of the intima, contributes to the lipid-rich necrotic core and fostering ECM degradation (11) and references therein). Finally, IFNγ-driven transition of SMCs from a contractile to a proliferative and migratory state is another important mark of atherosclerotic plaque progression (22). Multiple murine studies support the atheroma-promoting properties of IFNγ. LDL receptor (LDLr) and IFNγ double knockout (KO) showed much less atherosclerosis in the aortic arch and descending aorta than LDLr single KO mice after 8 weeks on a high fat diet (HFD) (12). Similarly, mice with double KO of ApoE and the IFN-γ receptor showed reduced aortic atherosclerotic lesion size compared to ApoE single KO mice after 3 months on a Western type diet (13). Myocardial rejection occurred in both wild-type and IFNγ-KO mice given heart transplants, but the IFNγ-KO mice were protected from developing coronary arteriosclerosis (14, 15). Wild-type mice treated with anti-IFNγ antibodies were also protected from atherosclerosis upon heart transplantation. Other murine studies have found that exogenous IFNγ administration greatly increases atherosclerotic lesion size while promoting MØ and T cell recruitment to the lesions (16).

MØ play a central role in atherosclerosis, from early foam cell formation to advanced plaque development and potential rupture. Studies have shown that the effect of MØ on atherosclerotic plaques is not only determined by the number of infiltrated macrophages but also by their polarization state and the relative proportion of different phenotypes. These cells exhibit remarkable plasticity, adopting various phenotypes in response to environmental cues within the atherosclerotic lesion. The heterogeneity of MØ populations in atherosclerotic plaques has been increasingly recognized, with distinct subsets showing pro-inflammatory, anti-inflammatory, or lipid-handling properties (17, 18). Recently, Mosquera et al. performed an integrative meta-analysis using single-cell RNAseq datasets from atherosclerotic lesions and non-lesion coronary arteries (7). This revealed disease-relevant MØ subtypes, including foamy MØ, monocytes, inflammatory MØ and tissue resident MØ in human atherosclerosis.

The interplay between MØ and IFNγ signaling is particularly relevant in the context of atherosclerosis. IFNγ can profoundly influence MØ function, promoting a pro-inflammatory phenotype and enhancing lipid uptake. Moreover, STAT1 activation in macrophages has been shown to exacerbate atherosclerosis in animal models, suggesting a critical role for this signaling axis in disease progression (4, 19–23). Indeed, Agrawal et al. identified STAT1 as an important regulator of foam cell formation and atherosclerotic lesion development in an intraperitoneal inflammation model and an atherosclerosis-susceptible bone marrow transplantation mouse model (22). Thus STAT1 was recognized to play a role in MØ apoptosis, a critical process for the formation of the necrotic core in atherosclerotic plaques (24). Mice transplanted with STAT1 deficient bone marrow revealed reduced MØ apoptosis and plaque necrosis (24). Silencing ARL11, ADP ribosylation factor like GTPase 11, was recently shown to relieve atherosclerotic inflammation in ApoEKO mice and lipid deposition in MØ via retraining JAK2/STAT1 pathway (25). Increased activity of STAT1 was also associated with decreased expression of contractile genes and as a consequence SMC de-differentiation (26), VSMCs proliferation and neointimal hyperplasia (15). Moreover, phosphorylated STAT1 in VSMCs and ECs of human atherosclerotic plaques correlated with elevated expression of the chemokines CXCL9 and CXCL10 (10).

In MØ, recent evidence supports a collaborative role of STAT1 with the Lineage Determining Transcription Factors (LDTF) PU.1 in MØ-specific transcriptional responses. PU.1 can bind cognate sequences in the context of closed chromatin and subsequently facilitate recruitment of Stimulus Dependent Transcription Factors (SDTF), like IFNγ-activated STAT1. Thus, PU.1 directs IFNγ-induced STAT1 to their genome-wide cognate binding sites in a cell type-specific manner to activate gene expression (9, 27, 28). This chromatin accessibility was also shown to be associated with enrichment of different histone acetylation and methylation marks, including H3K27Ac, H3K4me1 and H3K27me3, in gene promoters and enhancers (29, 30). Recent studies have also demonstrated that the alterations in histone acetylation and methylation patterns can significantly impact macrophage activation states and their contribution to plaque development (31, 32). Understanding how IFNγ and STAT1 signaling intersect with PU.1 and these epigenetic processes in MØ and MØ subtypes in atherosclerotic plaques could provide new insights into the molecular basis of MØ-specific STAT1-dependent transcription and its potential contribution to atherosclerosis.

Based on a comprehensive multi-omics approach, integrating ATACseq, ChIP-seq, and RNA-seq data from IFNγ-treated bone marrow-derived MØ, we identified a set of STAT1-dependent integrative genes that exhibit PU.1 co-binding combined with specific epigenetic and transcriptional characteristics. By using single-cell and bulk RNA sequencing data from human and mouse atherosclerotic lesions we were able to monitor the dynamic changes of these STAT1-dependent integrative genes in macrophage subtypes within atherosclerotic plaques.

Accordingly, we provide detailed insights into MØ-specific IFNγ-activated transcriptional changes, mediated by STAT1-PU.1 co-binding and associated epigenetic changes, and offer the identification of new biomarkers and therapeutic targets in atherosclerosis. Moreover, identification of a subset of 24 MØ-specific STAT1-dependent genes, commonly expressed in human and mouse atherosclerotic lesions, could represent a novel gene signature to monitor plaque progression during human atherosclerotic disease.

## Results

### Multi-omics based integration of MØ IFNγ-stimulated transcriptional changes in human and mouse atherosclerosis

**Figure 1:**
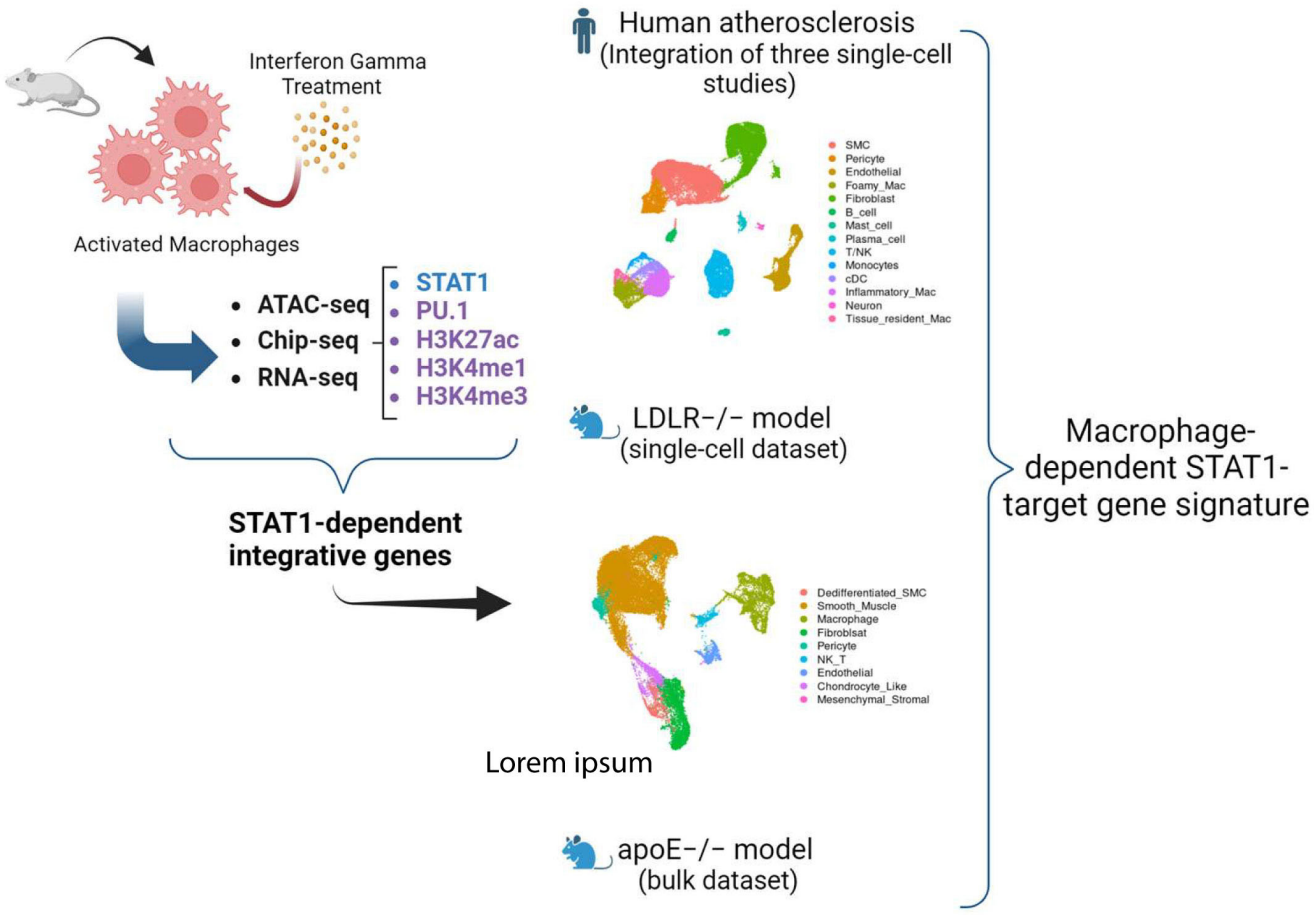
Schematic overview of the study. We performed a multi-omics strategy of different steps. First, we collected publicly available multi-omics data sets of short-term IFNγ treated mouse bone marrow-derived MØ, examining chromatin accessibility (ATAC-seq) and histone acetylation (H3K27ac) and methylation (ChIP-seq: H3K4me1 and H3K4me3) along with the chromatin interaction of key transcription factors (ChIP-seq) STAT1 and PU.1. We also included our in-house gene expression profiling dataset (RNA-seq) of mouse bone marrow-derived MØ treated with IFNγ for different time points. Subsequently, integration of these different data sets was performed to select a list of IFNγ-responsive STAT1-dependent integrative genes. Next, by using publicly available single-cell RNA-seq (scRNAseq) data sets of atherosclerotic lesions from human patients and of a LDLr-/- high fat diet (HFD) mouse model, we aimed at monitoring the dynamic changes of these STAT1-dependent integrative genes in macrophage subtypes within atherosclerotic plaques and identify novel diagnostic markers and therapeutic targets. Finally, a comparative analysis of these datasets enabled the identification of a novel MΦ-specific STAT1-dependent integrated gene signature for tracking human atherosclerotic plaque development.

In our quest to identify IFNγ-responsive STAT1-dependent integrated genes in MØ and understand their pathogenic and diagnostic behavior in atherosclerotic plaque formation, we performed a multi-omics strategy of different steps (**Figure 1**). First, we collected publicly available multi-omics data sets of short-term IFNγ treated mouse bone marrow-derived MØ, examining correlates of transcription activation, including chromatin accessibility (ATAC-seq) (33) and histone acetylation (H3K27ac) (28) and methylation (ChIP-seq: H3K4me1 and H3K4me3) (29) along with the chromatin interaction of key transcription factors (ChIP-seq) STAT1 (28, 34) and PU.1 (27) (**Supplementary Table S1**). To minimize batch effects, we prioritized selecting datasets that contained multiple correlates and were, whenever feasible, conducted by a single laboratory, followed by normalizing all the samples as described in materials and methods. We also included our in-house gene expression profiling dataset (RNA-seq) of mouse bone marrow-derived MØ treated with IFNγ for different time points (0, 0.5, 2, 4, 8, 24h) (**Supplementary Table S2**). Subsequently, integration of these different data sets was performed to select a list of IFNγ-responsive STAT1-dependent integrative genes based on the following criteria: IFNγ-induced 1) differential transcriptional activity, 2) chromatin accessibility, 3) epigenetic changes, 4) STAT1-PU.1 co-binding. Next, by using publicly available single-cell RNA-seq data sets of atherosclerotic lesions from human patients and of a LDLr-/- high fat diet (HFD) mouse model (**Supplementary Table S1**), we aimed at monitoring the dynamic changes of these STAT1-dependent integrative genes in MØ subtypes within atherosclerotic plaques and identify novel diagnostic markers and therapeutic targets. Finally, comparative analysis between these scRNAseq data sets and with our in-house HFD ApoE-/- atherosclerosis mouse model bulk RNA-seq data set (**Supplementary Table S1**) was used to identify a novel MØ-specific STAT1-dependent integrated gene signature to monitor human atherosclerotic plaque formation (**Figure 1**).

### Identification of IFNγ-responsive STAT1-dependent integrated genes

**Figure 2:**
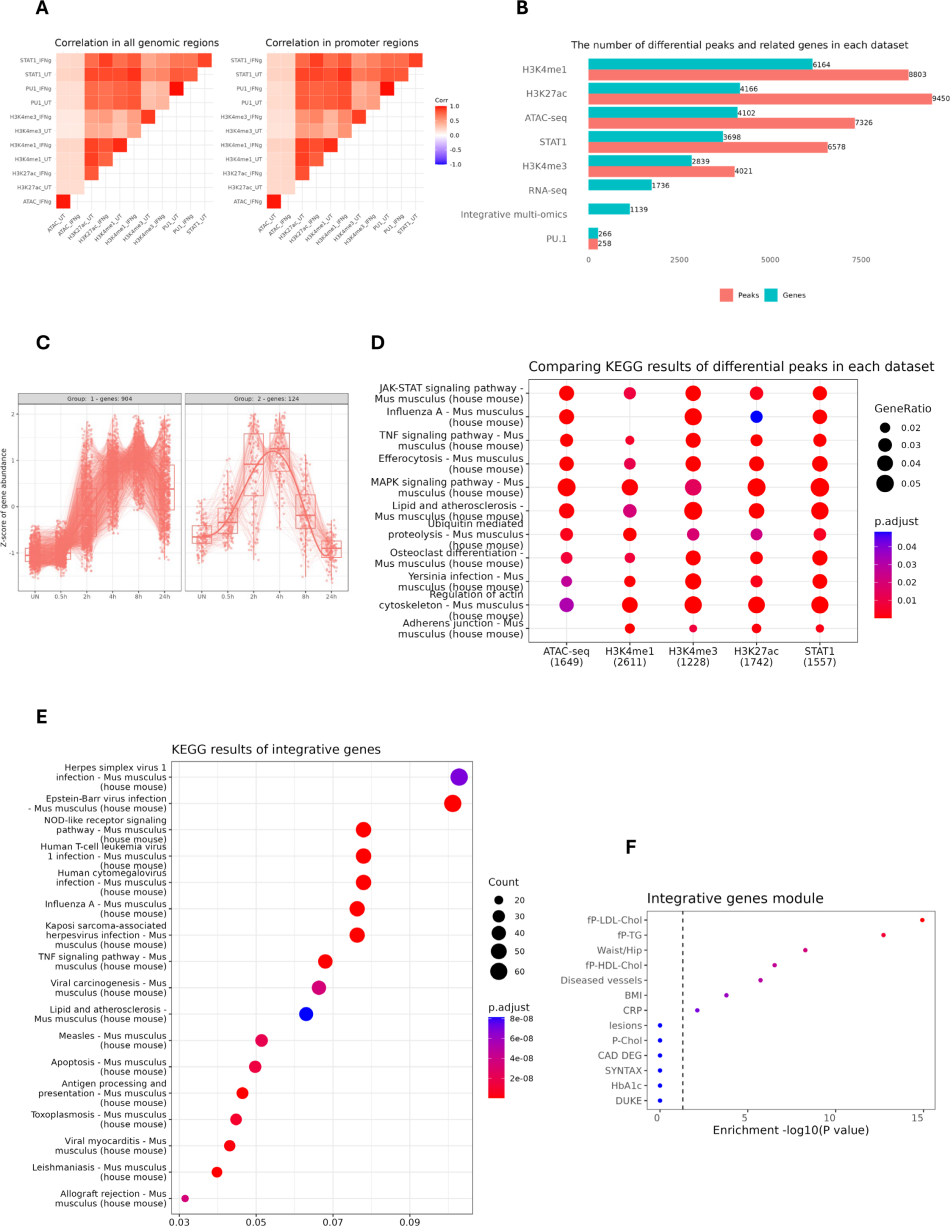
Identification and characterization of IFNγ-responsive STAT1-dependent integrated genes. **(A)** the pairwise correlation of the peaks derived from all genomic regions (left) and the peaks that are restricted to the promoter region (–3000, 3000). The color scale represents Pearson correlation coefficients, with red indicating high correlation and blue indicating low correlation across datasets. **(B)** The horizontal bar plot showing the number of differential peaks and their related genes (entrez gene id) in each dataset. For ATAC-seq, H3K4me1 & H3K4me3, a fold change cut-off of 2 was selected, whereas for PU.1, STAT1 and H3K27ac, a cut-off of 4 was set. The bars are sorted based on the number of genes in each dataset. **(C)** The expression pattern of STAT1-dependent integrative genes from our RNA-seq experiment performed on IFNγ-treated mouse MØ at different time points. X-axis denotes time points post-treatment; Y-axis shows normalized gene expression. Lines represent expression trends across key genes. **(D)** Kyoto Encyclopedia of Genes and Genomes **(KEGG)** pathway analysis of differential peaks for each dataset. The numbers in the parentheses indicate the total number of annotated genes in each dataset. The color scale represents the statistical significance of each enriched pathway with respect to its corresponding dataset. **(E)** KEGG pathway analysis of STAT1-dependent integrative genes. **(F)** The clinically phenotypic enrichment of integrative gene module using Stockholm-Tartu Atherosclerosis Reverse Network Engineering Task (STARNET) gene regulatory networks (26), indicating disease or trait associations. The color intensity represents the statistical significance of observed traits with red color indicating higher significance. fp-LDL-Chol, Fasting Plasma Low-Density Lipoprotein Cholesterol; fp-TG, Fasting Plasma Triglycerides; fp-HDL-Chol, Fasting Plasma High-Density Lipoprotein Cholesterol; BMI, Body Mass Index; CRP, C-Reactive Protein; P-Chol, Plasma Cholesterol; CAD DEG, Coronary Artery Disease Differentially Expressed Genes; SYNTAX, Synergy Between Percutaneous Coronary Intervention with Taxus and Cardiac Surgery; HbA1c, Hemoglobin A1c.

In the first step of our multi-omics analysis strategy, we integrated ATAC-seq and different ChIP-seq (STAT1, PU.1, H3K27ac, H3K4me1 and H3K4me3) datasets, based on peak calling and merging all the peaks. Next, we assessed global peak correlations across various datasets (**Figure 2A**). For this, we examined the pairwise correlation of the peaks derived from all genomic regions and peaks that were restricted to the promoter regions (–3000, 3000). As shown in **Figure 2A**, the correlation pattern looks similar regardless of the location of the peaks (all genomic regions *vs* promoter-exclusive regions). Moreover, we observed that increased STAT1 binding upon IFNγ exposure strongly correlated with enhanced H3K27ac and H3K4me1 marks. STAT1 binding also correlated with the presence of H3K4me3 marks and PU.1 chromatin interactions, but in a rather IFNγ-independent manner.

To identify IFNγ-responsive STAT1-dependent integrative genes, we performed differential peak analysis on the above normalized ATAC-seq and ChIP-seq datasets between control and IFNγ-treated conditions. Since PU.1 binding was only marginally affected by IFNγ treatment, we could hardly detect any differential peaks in comparison with STAT1 and different epigenetic marks. As such varying gene numbers for different data sets were identified, with the highest number for H3K4me1 (6164) and the lowest for H3K4me1 (2839). Then we integrated the differentially enriched regions from each data set with the list of 1736 IFNγ-induced genes derived from the RNAseq dataset of mouse MØ treated with IFNγ for different time points (0, 0.5, 2, 4, 8, 24h). This multi-omics integration resulted in the identification of 1139 IFNγ-responsive STAT1-dependent integrative genes (**Figure 2B**; **Supplementary Table S3**), of which cluster analysis identified two major gene expression patterns (**Figure 2C**). KEGG analysis of differential peaks in each dataset (except PU.1) highlighted relevant overlapping terms including JAK-STAT, MAPK and TNF signaling pathways and lipid and atherosclerosis (**Figure 2D**). Likewise, KEGG analysis for the IFNγ-responsive STAT1-dependent integrative genes recognized similar terms, especially connected to lipid and atherosclerosis (**Figure 2E**). Moreover, when we queried the Stockholm-Tartu Atherosclerosis Reverse Network Engineering Task (STARNET) gene regulatory networks across seven cardiometabolic tissues (35), the integrative genes highly associated with phenotypic traits such as LDL cholesterol and diseased vessels (**Figure 2F**).

### IFNγ-responsive integrated genes are characterized by STAT1-PU.1 co-binding in combination with increased histone methylation and acetylation and chromatin accessibility

**Figure 3:**
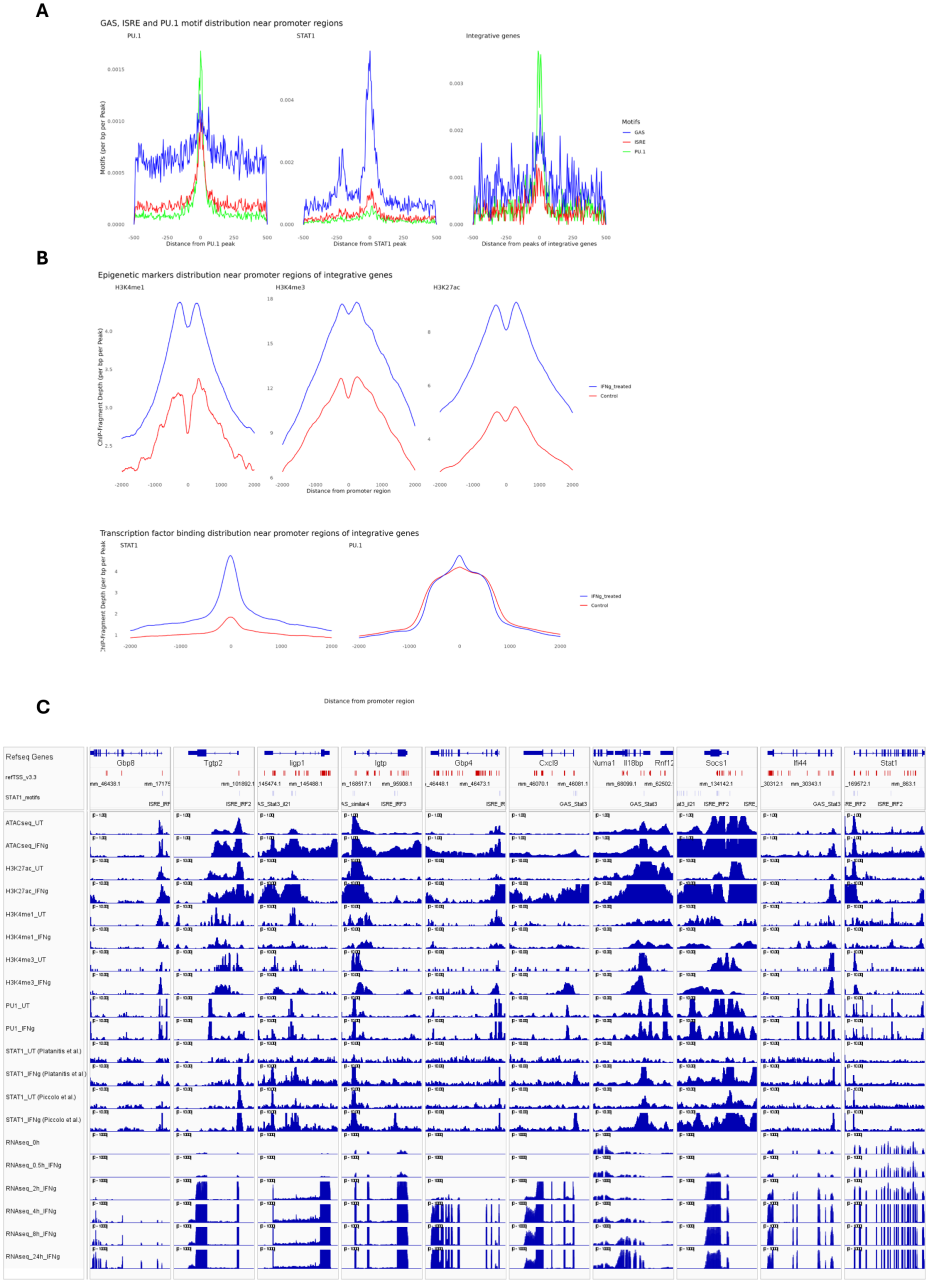
Epigenetic and binding profiles of IFNγ-responsive integrated genes. **(A)** The enrichment of PU.1 motif and GAS (Gamma-Activated Sequence) and ISRE (Interferon-Stimulated Response Element) motifs as binding sites for PU.1 and STAT1, respectively, in PU.1(left), STAT1(middle) datasets and in the integrative gene peaks (right). The PU.1 dataset (left) shows dominant enrichment of the PU.1 motif (blue), with minimal GAS or ISRE signals. The STAT1 dataset (middle) highlights strong enrichment of GAS (red) motif. The integrative gene peaks (right) display a balanced enrichment of all three motifs, indicating cooperative binding of PU.1 and STAT1 in IFNγ-responsive genes. **(B)** The methylation (H3K4me1/3), acetylation (histone H3K27ac), and transcription factor binding profiles (PU.1 and STAT1) of integrative genes within ±5 kb of promoter regions. **(C)** The transcriptional start sites (TSS), GAS and ISRE motifs coordinate as well as epigenetic, transcription factor binding and expression profiles of 10 integrative genes were visualized using Integrative Genomics Viewer (IGV). To ensure the consistency of STAT1 binding pattern in response to IFNγ, we showed STAT1 peaks from two separate studies. TSS is marked as red vertical lines, while GAS and ISRE motifs were marked as gray vertical lines, showing STAT1 binding sites. All datasets are normalized to ensure comparability, with genomic coordinates aligned to the mouse genome (GRCm38/mm10).

Further characterization of IFNγ-responsive STAT1-dependent integrative genes included analysis of the correlation between STAT1 and PU.1 chromatin interactions and H3K27ac, H3K4me1 and H3K4me3 histone modifications upon IFNγ treatment as compared to control. For this, we performed promoter analysis of the 1139 IFNγ-responsive STAT1-dependent integrative genes for the presence of PU.1 and STAT1 binding sites (**Figure 3A**). We detected single or combined PU.1, GAS and/or ISRE motifs in the promoters of most of these genes, with GAS favoring ISRE for STAT1 binding. As observed in **Figure 3A**, GAS, ISRE and PU.1 motifs were highly enriched in the promoters of these genes. Moreover, the localized distribution of STAT1 and PU.1 motifs correlated with STAT1-PU.1 co-binding near the transcription start site (TSS) of IFNγ-responsive STAT1-dependent integrative genes. In general, under these conditions GAS motifs correspond to potential binding of GAF and ISRE motifs to ISGF3. In this context, the histone methylation (H3K4me1, H3K4me3) and acetylation (H3K27ac) of integrative gene promoters displayed a bimodal pattern, flanking STAT1 and PU.1 binding sites at TSS, and increased in response to IFNγ treatment as compared to control (**Figure 3B**). As already mentioned above, PU.1 binds DNA already in untreated conditions and does not change significantly in response to IFNγ stimulation (**Figure 3B**). To obtain a multi-omics perspective of the IFNγ-dependent distribution of STAT1, PU.1, H3K27ac, H3K4me1 and H3K4me3 binding in relation to chromatin accessibility and transcriptional activity of integrative genes, we prepared an Integrative Genomics Viewer (IGV) snapshot of 10 pre-selected IFNγ-responsive STAT1-dependent integrative genes (**Figure 3C**). These included Gbp8, Tgtp2, Ligp1, Igtp, Gbp4, Cxcl9, Il18bp, Socs1, Ifi44 and Stat1. For transcriptional start site (TSS) location of these genes (upper tracks), we used the mouse refTSS (36) annotated reference dataset. Besides, we determined the genomic coordinates of GAS and ISRE motifs present in their promoters, reflecting GAF and ISGF3 binding respectively. Based on this multi-omics examination of selected STAT1-dependent integrative genes, it was evident that chromatin is physically accessible for the binding of PU.1 in untreated conditions and STAT1 together with PU.1 upon exposure to IFNγ, particularly near the promoter regions surrounding TSS. This differential PU.1-STAT1 co-binding clearly correlated with histone H3K27ac marks around STAT1 binding sites, already present in untreated cells and highly enriched upon IFNγ treatment, as well as with active transcription. A similar association could be observed with dynamic changes of H3K4me1 and H3K4me3 marks (**Figure 3C**), with H3K4me3 concentrated near the TSS and H3K4me1 flanking these regions (37, 38). Together, these observations agree with a mechanism in which prior to IFNγ treatment, the chromatin/promoters of these integrative genes are in a poised or already active (constitutive) state, characterized by constitutive PU.1 binding (and to a lesser extent STAT1) in combination with histone methylation (both H3K4me1 and H3K4me3) and acetylation (H3K27ac) marks. But upon IFNγ exposure, there is a surge in chromatin openness, mediated by increased histone acetylation along with increased STAT1 co-binding (in the form of GAF and ISGF3) to sites pre-bound by PU.1 that accelerates transcriptional activation and marks these genes as IFNγ-responsive STAT1-dependent integrative genes.

### STAT1-integrative genes display MØ subtype-dependent expression in human atherosclerotic plaques

**Figure 4:**
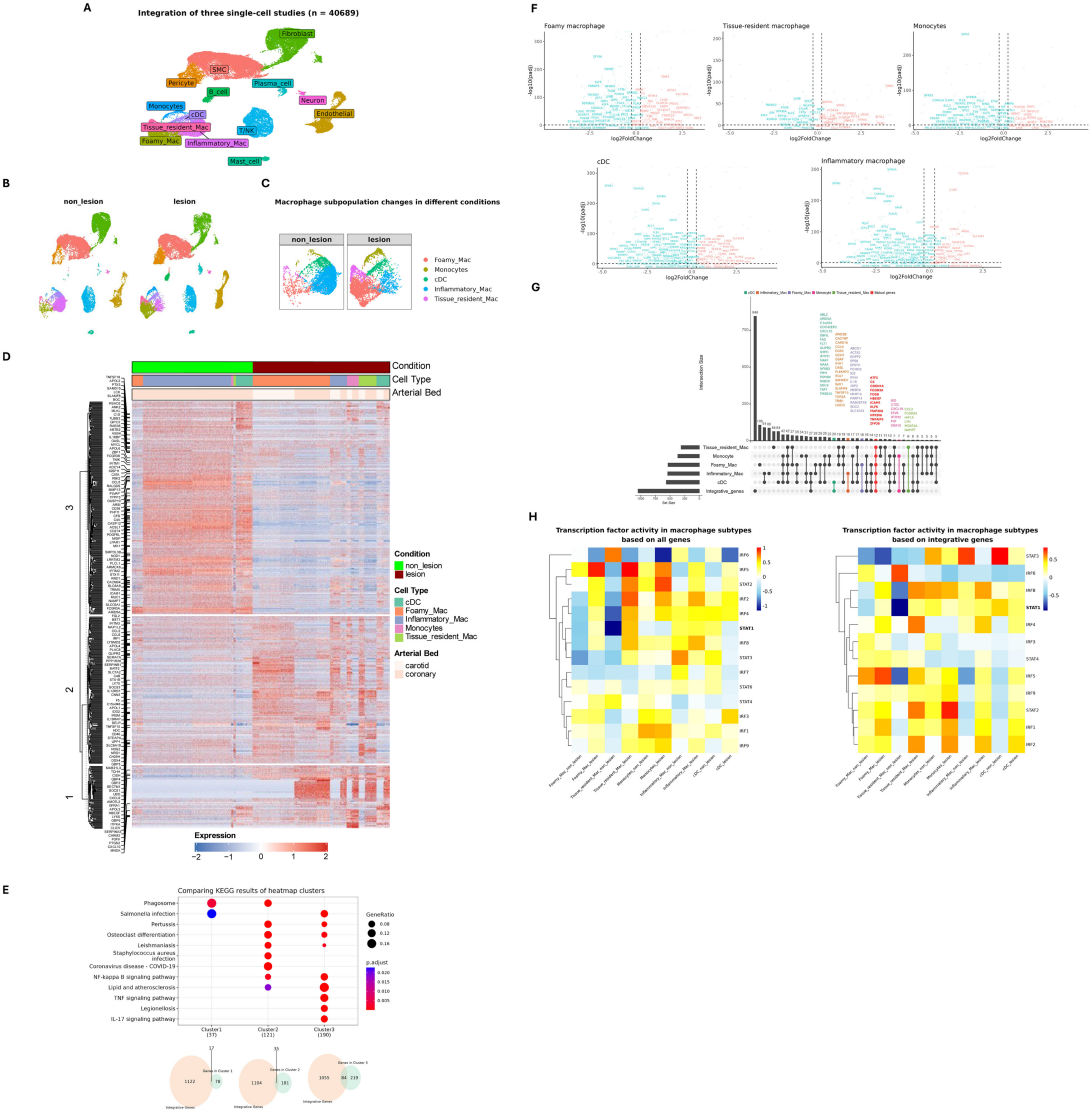
MØ subtype-dependent expression of STAT1-target genes in human atherosclerotic plaques. **(A)** The Uniform Manifold Approximation and Projection (UMAP) projection of the three single-cell RNA-seq studies with major cell annotations (See **Supplementary Table S1** for sample details). Each point represents a single cell, colored according to its annotated cell type. **(B)** Comparing lesion *vs* non-lesion groups showed dynamic changes among various MØ subtypes. The number of cells in non-lesion and lesion group were 20666 and 20023, respectively. **(C)** The number of foamy MØ, monocyte and tissue resident MØ increased in the lesion group. **(D)** The heatmap representing the expression profile of differentially expressed genes (non-lesion *vs* lesion) in MØ population with respect to cell type and arterial bed. The hierarchical cluster analysis generated three distinct clusters. The rows represent individual DEGs identified in the MØ population. STAT1-target genes are annotated on the left side of the heatmap to highlight their relevance in the context of MØ function or lesion development. **(E)** KEGG pathway analysis of each cluster showed cluster-specific signaling pathways. The intersection of each Venn diagram shows the number of integrative genes (STAT1-target genes) in each cluster (Cluster 1, Cluster 2, and Cluster 3). **(F)** The expression pattern of differentially expressed genes (lesion *vs* non-lesion) in various macrophage subtypes. The STAT1-target genes were labeled in each dot plot. |log2FoldChange| >= 0.25 was used as the cut-off; FDR < 0.05. Red and blue colors represent up-regulated and down-regulated genes, respectively. **(G)** UpSet plot showing cell type-specific integrative gene sets. The sets on the left (except for integrative genes) indicate the number of cell type-specific differentially expressed genes. The red-colored set shows the unique STAT1-dependent genes present in all MØ subtypes. Bars on the right indicate the size of gene set intersections, with each bar corresponding to a specific combination of macrophage subtypes (shown by filled circles below the bar). **(H)** The assessment of transcription factor (TF) activity of STAT and IRF family in diverse macrophage subtypes based on all genes (left) providing a broad view of regulatory patterns, or integrative genes (right) highlighting TFs critical to regulation of integrative genes. For comparison purposes, STAT1 is bolded. The hierarchical clustering on the left side shows TFs with similar activities.

To further understand the pathogenic and diagnostic behavior of IFNγ-responsive STAT1-dependent integrative genes in macrophage subtypes in atherosclerotic plaque formation, we examined several publicly available single-cell RNA-seq data sets of atherosclerotic lesions from human patients and of a LDLr-/- HFD mouse model (**Supplementary Table S1**). In the context of human atherosclerosis, two human datasets from coronary and carotid atherosclerotic lesions and one dataset from non-lesion coronary arteries were used (see **Supplementary Table S1** for the detailed sample description). The latter data set included material obtained from 3 patients with end-stage heart failure undergoing cardiac transplantation, without arterial anomaly (39). We integrated these three single cell studies, followed by cell type annotation using a combination of automated and manual approaches, consisting of 40689 cells (**Figure 4A**; **Supplementary Table S4**). The rapid advances in single-cell technologies have facilitated the identification of diverse MØ subtypes, based on expression of specific markers for pro-inflammatory MØ (TNF, CXCL2), foamy anti-inflammatory MØ (TREM2, CD9) and resident-like MØ (FOLR2, CBR2), detected in atherosclerotic plaques in both human and mouse (40–42). Consistent with Mosquera et al., 2023 (7), we identified foamy MØ, monocytes, inflammatory MØ, tissue resident MØ and conventional dendritic cells (cDC) in both non-lesion and lesion groups. Comparing lesion *vs* non-lesion groups displayed dynamic changes among various MØ subtypes. For instance, the number of foamy MØ, monocyte and tissue resident MØ increased in lesion group (**Figures 4B, C**), whereas inflammatory MØ numbers were higher in non-lesion group *vs* lesion. Moreover, differential expression analysis (selection criteria: FDR < 0.05 and log2FC >= 0.25 and log2FC <= -0.25) identified 614 genes that were differentially expressed in non-lesion *vs* lesion group in all MØ subtypes combined.

To obtain a holistic view of the expression pattern of these differentially expressed genes, we prepared a heatmap showing the expression pattern with respect to MØ subtype and arterial bed (**Figure 4D**; **Supplementary Table S5**). We then performed hierarchical cluster analysis using the Euclidean method, generating three distinct clusters. Interestingly, clusters 1 and 2 unveiled arterial bed-specific gene expression patterns, with genes in cluster 1 predominantly upregulated in coronary plaque tissue and present in all MØ subtypes. In contrast, genes in cluster 2 were associated with carotid lesion expression, again detected in all MØ subtypes. Cluster 3 represented genes with MØ subtype-specific gene expression, depending on the condition and vascular bed. For example, high expression of these genes in non-lesion coronary arteries was mainly associated with inflammatory MØ, monocytes and cDCs. On the other hand, expression in tissue resident MØ was higher in carotid lesions as compared to coronary lesions, whereas foamy MØ displayed barely detectable levels in both non-lesion and lesion samples. KEGG enrichment analysis highlighted the unique characteristics of cluster 1, 2 and 3 genes and their relation to atherosclerosis (**Figure 4E**). Indeed, Cluster 1 genes were primarily associated with phagocytosis, whereas genes in cluster 2 and 3 were more connected to innate immunity, inflammatory response and lipid metabolism.

Among the differentially expressed genes shown in **Figure 4D**, 136 STAT1-dependent integrative genes could be recognized, especially in cluster 3 and 2 and to a lesser extent in cluster 1 (**Figures 4D, E**). This implied that subsets of STAT1-dependent integrated genes behave as general MØ markers or are expressed in a more MØ subtype-dependent manner associated with arterial bed. Following their differential expression (lesion *vs* non lesion: FDR < 0.05 and log2FC >= 0.25 and log2FC <= -0.25) in specific MØ subtypes identified multiple expression characteristics, which clearly correlated with cluster 2 and 3 behavior (**Figure 4F**). For example, high expression of subsets of STAT1-dependent integrated genes in inflammatory MØ, monocytes and cDCs present in non-lesion arteries correlated with predominant downregulation in lesions. On the other hand, expression analysis in resident MØ and foamy MØ identified both up and downregulated STAT1-dependent integrated gene subsets, agreeing with high expression observed in arterial lesions (**Figure 4D**).

Using DoRothEA collection, a curated dataset of Transcription Factors and their transcriptional targets, further validated STAT1 activity in these different macrophage sub-types (43) (**Figure 4H**). Close examination of STAT1 activity on either 22385 genes or only 1009 STAT1-integrative genes revealed increased activity in both foamy MØ and tissue resident MØ present in lesion arteries. In inflammatory MØ, monocytes and cDCs, STAT1 activity appeared higher in non-lesion arteries, which correlated with high expression of subsets of STAT1-dependent integrated genes in these sub-types. Interestingly, this MØ sub-type dependent STAT1 activity, was also observed for STAT2, IRF4, IRF5, IRF8 and IRF9 (**Figure 4H**).

To provide more insight into the MØ subtype-dependent behavior of STAT1-dependent integrated genes, we grouped genes according to their expression behavior. As such, we identified subsets of STAT1-dependent integrated genes that were specifically expressed in tissue resident MØ, monocytes, foamy MØ, inflammatory MØ and cDCs. Also, a group of genes could be recognized that were commonly expressed in all MØ subtypes and included ATF3, C3, CDKN1A, FCGR3A, FOSB, HBEGF, ICAM1, KLF6, MAP3K8, NFKBIA, TNFAIP3 and ZFP36 (**Figure 4G**, genes in red), with known inflammation and atherosclerosis-related functions. Therefore, MØ-dependent expression of STAT1-dependent integrated genes can serve as general MØmarkers or are expressed in a more MØ subtype-dependent manner in human atherosclerotic plaques.

To further substantiate this MØ-dependent nature, we also assessed the expression profile of STAT1-dependent integrated genes among differentially expressed genes (non-lesion *vs* lesion) in the vascular smooth muscle cell (VSMCs) population (**Supplementary Figure S1A**). First, hierarchical clustering identified 511 differentially expressed genes in VSMCs divided over three clusters and correlated with arterial bed-specificity. Similar to MØ (**Figure 4D**), clusters 1 and 2 unveiled arterial bed-specific gene expression patterns, with genes in cluster 1 predominantly upregulated in coronary plaque tissue as compared to the non-lesion group. In contrast, genes in cluster 3 were associated with higher carotid lesion expression. Finally, cluster 2 represented genes already highly expressed in non-lesion material. Interestingly, in each cluster different VSMC subpopulations seemed to be present (**Supplementary Figure S1A**), implying that genes are expressed in a VSMC subtype-dependent manner associated with arterial bed. KEGG enrichment analysis highlighted the unique characteristics of cluster 1, 2 and 3 genes and their relation to VSMC function and atherosclerosis (**Supplementary Figure S1B**). Indeed, Cluster 1 genes were primarily associated with immune response, whereas genes in cluster 2 and 3 were more connected to cytoskeleton changes and contraction (**Supplementary Figure S1B**). Among the VSMC-dependent differentially expressed genes shown in **Supplementary Figure S1A**, 117 STAT1-dependent integrative genes could be recognized, especially in cluster 3 and 2 and to a lesser extent in cluster 1 (**Supplementary Figure S1B**).

### MØ sub-type dependent expression of STAT1-integrative genes in mouse aortic plaques identifies overlap with human atherosclerosis

**Figure 5:**
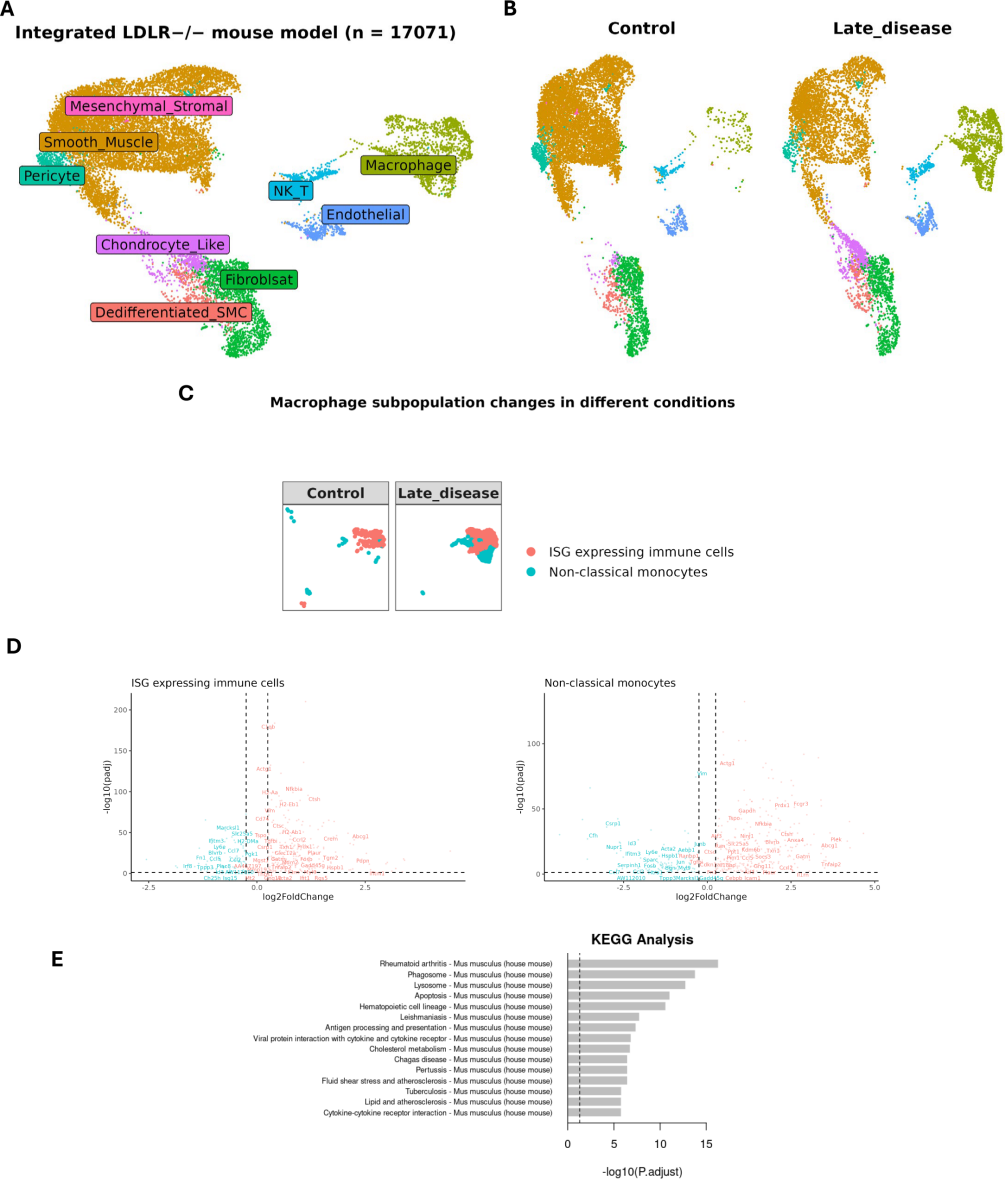
Expression profile of MØ-dependent STAT1-target genes in LDLR knockout mouse model. **(A)** UMAP projection of an integrated single-cell RNA-seq study consisting of 17071 cells (See **Supplementary Table S1** for sample details). Each point represents a single cell. colored by cell type. **(B)** Comparing late disease *vs* control groups revealed changes in the relative abundance or proportion of cell populations, **(C)** particularly in MØ subtypes namely ISG-expressing immune cells and non-classical monocytes, reflecting disease-driven immune cell remodeling. **(D)** The expression pattern of differentially expressed genes (late disease *vs* control) in various MØ subtypes. The STAT1-target genes were labeled in each dot plot. |log2FoldChange| >= 0.25 was used as the cut-off; FDR < 0.05. Red and blue colors represent up-regulated and down-regulated genes, respectively. **(E)** KEGG pathway analysis for 400 differentially expressed genes in mouse single cell dataset (late disease *vs*. control). The x-axis represents the statistical significance of enrichment. The y-axis lists the top enriched KEGG pathways.

In order to compare MØ-dependent STAT1-integrative gene expression between human and mouse atherosclerosis, we also analyzed a single-cell RNA-seq data set of aortic lesions from a LDLr-/- HFD mouse model, comparing LFD (Control) *vs* HFD (12 weeks: Late Disease) (**Figure 5A**; **Supplementary Table S1** (8). Integrative analysis combined with cell type annotation selected 17071 cells, and revealed dynamic changes in various cell populations, with a clear increase in MØ in Late Disease (**Figure 5B**). We further focused on the MØ population and annotated ISG-expressing immune cells and non-classical monocytes based on the specific markers (IFIT1, IFIT2, IFIT3, IFIT5, ISG15, CCL3, CCL4, CCL3L3, RSAD2, OASL, CXCL10, IFI15, ISG20) and (CD14, CD16, CD11b, CD68, HLA-DR, CD33, CD11c, CD123, CD15, CD3D, CD3E, CD3G, CD3Z, CD66b, FCGR3A, CDKN1C, LST1, FCER1G, MS4A7, RHOC, S100A8, S100A9, CST3, C1QC), respectively (44) (**Figure 5C**). ISG-expressing immune cells generally display inflammatory characteristics (45). However, non-classical monocytes often show more anti-inflammatory properties (46). It should also be noted that there is currently no consensus on the number of MØ subtypes in mice and due to using different experimental protocols, disparities exist in detection of multiple MØ subtypes (47). When comparing Late disease *vs* control groups, both ISG-expressing immune cells and non-classical monocytes displayed higher numbers in the lesion group (**Figure 5C**), Moreover, differential expression analysis (selection criteria: FDR < 0.05 and log2FC >= 0.25 and log2FC <= -0.25) identified 400 genes that were differentially expressed in non-lesion *vs* lesion group in both MØ subtypes. Among these differentially expressed genes, 73 STAT1-dependent integrative genes could be recognized, with their lesion-dependent changes in ISG-expressing immune cells and non-classical monocytes shown in **Figure 5D**. KEGG enrichment analysis highlighted the relation of these 400 genes to immunity and atherosclerosis and functional overlap with differentially expressed genes in MØ sub-types of human atherosclerotic lesions (**Figure 5E**).

**Figure 6:**
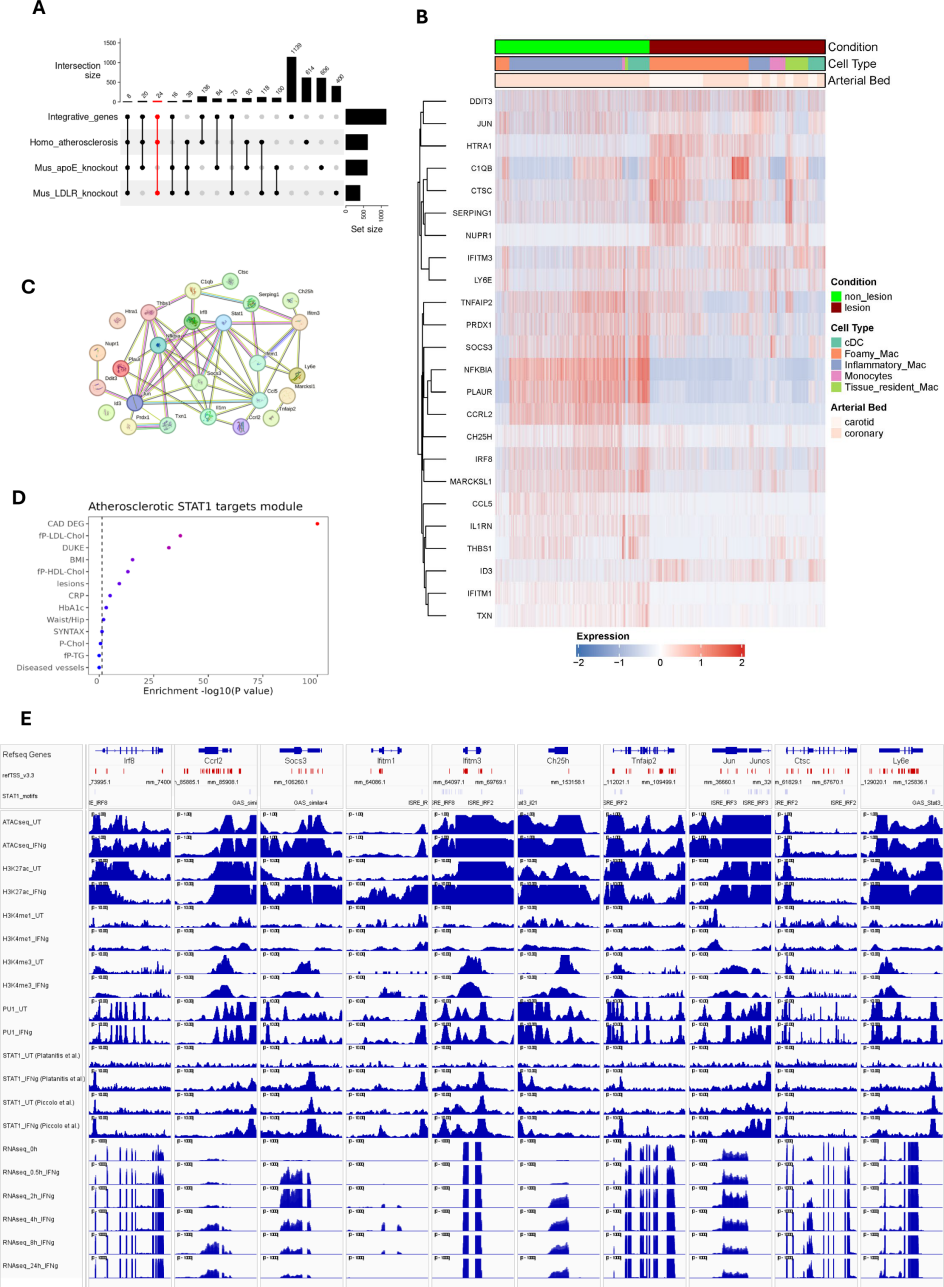
STAT1-dependent gene signature in Atherosclerosis progression. **(A)** UpSet plot demonstrates the number of integrative genes across mice and human models. The sets on the right (except for integrative genes) indicate the number of dataset-specific differentially expressed genes. Vertical bars represent the number of DEGs unique to each dataset (e.g., human-specific, mouse-specific) or shared across datasets, with numbers indicating gene counts for each combination. Dots and connecting lines below the bars denote which datasets contribute to each intersection. The red-colored set shows 24 STAT1-integrative gene set, specifically expressed in MØ sub-types across human and mouse atherosclerotic lesions (LDLR-/- HFD). **(B)** The expression pattern of these 24 genes were traced in human atherosclerosis. **(C)** The protein-protein interaction network of 24 macrophage-dependent STAT1-target gene signature, prepared using STRING database. Lines connecting nodes indicate protein-protein interactions, with thickness reflecting the confidence score (strength of data support) from STRING. **(D)** The clinical traits that were associated with 24 gene signatures based on STARNET database, as depicted in **Figure 2F**. Traits with strong associations (red color) are likely influenced by the STAT1-driven gene signature. The color intensity represents the statistical significance of observed traits. **(E)** The epigenetic and transcriptomic profiles of 10 select signature genes, as described in **Figure 3C**.

Subsequent, comparative analysis between atherosclerotic lesions from human patients and of a LDLr-/- HFD mouse model, identified subsets of overlapping and unique MØ-dependent genes. For example, human plaques showed 614 differentially expressed macrophage-dependent genes, which included 136 STAT1-integrative genes (**Figure 6A**). Likewise, 400 differentially expressed MØ-dependent genes were present in mouse lesions, including 73 STAT1-integrative genes. Interestingly, comparative analysis identified 118 genes commonly expressed in MØ sub-types across human and mouse atherosclerotic lesions (LDLR-/-), amongst which were 24 STAT1-integrative genes (**Figure 6A**). This highlights the overlap between human atherosclerosis and mouse atherosclerosis models and the potential involvement of STAT1-integrative genes in a MØ-dependent manner.

We also included a second mouse atherosclerosis data set, in which we performed bulk RNA-seq on aorta from high-fat diet (HFD) fed ApoE knockout mice to identify 606 HFD-differentially expressed genes (padj<0.05 and |log2FC| > 1) (**Supplementary Table S6**) (48). Comparative analysis between atherosclerotic lesions from the ApoE-/- and of a LDLr-/- HFD mouse models, identified a subset of 100 common genes, amongst which 16 STAT1-integrative genes with a MØ-dependent character (**Figure 6A**). Likewise, comparing atherosclerotic lesions of the ApoE-/- HFD mouse model and from human patients, identified 93 overlapping genes with 20 being STAT1-integrative and MØ-dependent (**Figure 6A**). Finally, 39 commonly expressed genes, including 8 STAT1-integrative genes, were identified in atherosclerotic plaques from human patients and ApoE-/- and LDLr-/- high fat diet mouse models.

### Identification of a STAT1-dependent gene signature in human atherosclerosis progression

Based on the above-described comparative analysis, we aimed at identifying a novel MØ-specific STAT1-dependent gene signature that could serve to monitor human atherosclerotic plaque formation (**Figure 1**). Accordingly, we selected 24 STAT1-integrative gene set, specifically expressed in MØ sub-types across human and mouse atherosclerotic lesions (LDLR-/- HFD) (**Figure 6A**). These genes included Ccl5, Ccrl2, Ctsc, Ddit3, Htra1, Id3, Ifitm3, Jun, Ly6e, Marcksl1, Nfkbia, Nupr1, Plaur, Prdx1, Serping1 and Txn1, shared between atherosclerotic lesions from human patients and of the LDLr-/- HFD mouse model. A remaining group of 8 genes, C1qb, Ch25h, Ifitm1, Il1rn, Irf8, Socs3, Thbs1, Tnfaip2, were also expressed in aorta from the ApoE-/- HFD mouse model (**Figure 6A**).

Characterization of these genes in the literature indeed revealed close connections to inflammation and atherosclerosis.

To further evaluate the behavior of these genes in human atherosclerosis, we prepared a heatmap showing the expression pattern with respect to MØ subtype and arterial bed (**Figure 6B**). After hierarchical cluster analysis, two different clusters could be recognized, dividing genes according to their expression profile. The expression of genes in cluster 1, including DDIT3, JUN, HTRA1, C1QB, CTSC, SERPING1, NUPR1, IFITM3 and LY6E appeared especially higher in carotid lesions as compared to coronary lesions, and restricted to foamy MØ, tissue resident MØ, monocytes and cDC. On the other hand, genes in cluster 2, TNFAIP2, PRDX1, SOCS3, NFKBIA, PLAUR, CCRL2, CH25H, IRF8, MARCKSL1, CCL5, IL1RN, THBS1, ID3, IFITM1, TXN, were characterized by higher expression in non-lesion coronary arteries and mainly associated with inflammatory MØ, monocytes and cDCs.

Using STRING database, a protein-protein interaction network was constructed (49). The majority of these 24 genes unveiled functional and physical associations with STAT1 acting as a hub (**Figure 6C**). Besides, STARNET analysis indeed connected these genes to phenotypic traits such as cardiovascular diseases, cholesterol and lesions (**Figure 6D**). Also, active transcription of a selection of these 24 signature genes coincided with prominent promoter STAT1-PU.1 co-binding, with the presence of GAS and/or ISRE sites representing potential GAF and ISGF3 binding. Moreover, increased histone methylation and acetylation and chromatin accessibility (**Figure 6E**), confirms characteristics of macrophage-dependent STAT1-integrative genes (**Figure 3C**).

Together, this confirms the important role of macrophage-dependent STAT1-driven transcription in atherosclerotic plaque formation and specifically identifies a STAT1-dependent gene signature that could help monitor plaque progression in human atherosclerotic disease. To determine a potential association of the expression of these genes with IFNγ produced in the plaque environment, we prepared feature plots derived from scRNA-seq datasets, illustrating the expression levels of IFNγ in NK and T cell (NK/T) populations for human (left panel) and mouse (right panel) control and lesion samples (**Supplementary Figure S2**). Obviously, in both species the number of IFNγ expressing NK/T cells (colored in blue) was very low as compared to non-expressing cells (grey), with the expression in human cells tending to be higher in non-lesion *vs* lesion. In contrast, in the mouse, the number of IFNγ-positive NK/T cells was too low to draw further conclusions (**Supplementary Figure S2**). Likewise, in the ApoE-/- HFD mouse model RNAseq data set, the transcript for *ifng* was below the detection level (not shown).

## Discussion

This study investigated the role of MØ STAT1-mediated signaling in atherosclerosis progression through multi-omics integration of IFNγ-induced MØ and expression analysis in human and mouse atherosclerotic lesions. First, by integrating ATAC-seq, ChIP-seq, and RNA-seq data from IFNγ-treated and untreated bone marrow-derived MØ, we identified 1139 STAT1-dependent integrative genes. Collectively, active transcription of these IFNγ-responsive STAT1-integrative genes was characterized by prominent promoter STAT1-PU.1 co-binding, increased histone methylation and acetylation and chromatin accessibility. Our observations predict a mechanism in which prior to IFNγ treatment the chromatin/promoters of these STAT1-integrative genes are in a poised or already active (constitutive) state, characterized by constitutive PU.1 binding (and to a lesser extent STAT1) in combination with histone methylation (both H3K4me1 and H3K4me3) and acetylation (H3K27ac) marks. But upon IFNγ exposure, there is a surge in chromatin openness, mediated by increased histone acetylation along with augmented STAT1 co-binding, reflecting binding of GAF and ISGF3, to sites pre-bound by PU.1 that accelerates transcriptional activation and marks these genes as IFNγ-responsive STAT1-dependent integrative genes. This is in agreement with current models, in which TFs activated by MØ stimulation and polarization with a canonical inflammatory agent (IFNγ, lipopolysaccharide [LPS]), such as STAT1, NF-kB and IRF1, have shown to land at regulatory elements pre-defined by PU.1 and constitutively marked by H3K4me1 (27, 28, 50, 51). Under these conditions, constitutive and poised states have been recognized, based on the presence or absence of basal histone acetylation (29). By investigating the genome-wide distribution of IRF1, IRF8, STAT1, and PU.1 in chromatin from resting and from IFNγ-activated MØ, Langlais and colleagues proposed a mechanism of constitutive chromatin co-binding of IRF8 together with PU.1, and to a lesser extent STAT1, to maintain basal H3K27 acetylation and target gene expression. But upon IFNγ exposure, an increase in histone acetylation along with increased STAT1 co-binding to sites pre-bound by PU.1 and IRF8 accelerates transcriptional activation of target genes (27). This coincided with increased IRF1 expression and recruitment of IRF1 together with IRF8, STAT1, and PU.1. The resulting transcriptional program is also marked as the IRF8/IRF1 regulome.

The co-binding of STAT1 and PU.1 along with histone methylation and acetylation at STAT1-integrative genes promoters in IFNγ-treated and untreated MØ, as observed in our multi-omics analysis, aligns with these findings and other studies that emphasize the importance of transcription factor interactions together with epigenetic changes in regulating inflammatory gene expression in MØ (52, 53). It also supports a role of PU.1 in cell-type specific IFNγ-induced STAT1-dependent gene expression in MØ. The nature of the genome-wide PU.1-STAT1 collaboration and the involvement of cell type-unique epigenetic marks in this respect is currently not known.

Thus in the context of MØ activation and M1 polarization, STAT1-containing complexes ISGF3 and GAF have shown to collaborate with PU.1, NF-kB and multiple IRFs to enhance transcriptional regulation of many pro-atherogenic genes, connected to stress, immune and inflammatory response, response to cytokine, regulation of cell proliferation and migration, regulation of cell adhesion and chemotaxis, cell death and apoptotic process, response to lipid, and reactive oxygen species (ROS) (9, 22). This is in line with the KEGG-analysis of our IFNγ-responsive STAT1-dependent integrative genes, which predominantly linked these genes to lipid metabolism and atherosclerosis-related pathways, whereas STARNET analysis identified high association with LDL cholesterol and diseased vessel traits. To further understand the pathogenic and diagnostic behavior of IFNγ-responsive STAT1-dependent integrative genes in MØ subtypes in human atherosclerotic plaque formation, two human single cell RNAseq datasets from coronary and carotid atherosclerotic lesions and one dataset from non-lesion coronary arteries were used. In agreement with Mosquera and colleagues (7), we were able to distinguish foamy MØ, monocytes, inflammatory MØ, tissue resident MØ and conventional dendritic cells (cDC) in both non-lesion and lesion groups (54). Also, the number of foamy MØ, monocyte and tissue resident MØ increased in lesion group, whereas inflammatory MØ numbers were higher in non-lesion group *vs* lesion (7). Moreover, 614 genes were differentially expressed in non-lesion *vs* lesion group in any of the MØ subtypes, among which 136 could be recognized as STAT1-dependent integrated genes. Hierarchical clustering analysis unveiled arterial bed-specific and MØ-subtype dependent expression patterns of these genes, whereas KEGG enrichment analysis highlighted their unique characteristics and their relation to inflammation and atherosclerosis. More in depth analysis revealed dynamic changes of STAT1-dependent integrated genes in MØ subtypes. For example, high expression of subsets of STAT1-dependent integrated genes in inflammatory MØ, monocytes and cDCs present in non-lesion arteries correlated with predominant downregulation in lesions. On the other hand, expression analysis in resident MØ and foamy MØ identified both up and downregulated STAT1-dependent integrated gene subsets, agreeing with high expression observed in arterial lesions. Interestingly, this correlated with MØ sub-type dependent STAT1 activity, as well as for STAT2, IRF4, IRF5, IRF8 and IRF9, and agrees with a known role of these TFs in atherosclerosis (55–59), suggesting the transcriptional collaboration of STAT1 with STAT2 and IRF family members, in the form of GAF, ISGF3 (STAT1,STAT2 and IRF9) and multiple IRFs, in these MØ subtypes.

Among subsets of STAT1-dependent integrated genes we identified MØ subtype common and specific genes, implying that STAT1-dependent integrated genes can serve as general MØ markers or are expressed in a more MØ subtype-dependent manner in human atherosclerotic plaques. In this respect, a group of STAT1-dependent integrated genes commonly expressed in all MØ subtypes included ATF3, C3, CDKN1A, FCGR3A, FOSB, HBEGF, ICAM1, KLF6, MAP3K8, NFKBIA, TNFAIP3 and ZFP36. ATF3 is a key transcription factor involved in regulation of inflammation and lipid metabolism in macrophages (60). Buono and colleagues reported that disrupted C3 (Complement C3) affects atherosclerosis progression (61). Cyclin-dependent kinase inhibitor CDKN1A is involved in inducing cellular senescence in MØ, contributing to atherosclerosis progression by releasing pro-inflammatory factors (62). NFKBIA is shown to be linked with coronary artery disease in the Chinese population (63). ICAM1, MAP3K8 and TNFAIP3 are highly associated with the development of atherosclerosis (64–66). FOSB and HBEGF are reported to be upregulated in atherosclerotic plaques (67, 68). KLF6 appears to be a key regulator of MØ inflammatory responses in the context of atherosclerosis. It mainly promotes pro-inflammatory activation and gene expression while suppressing anti-inflammatory pathways (69) while ZFP36 is engaged in inhibiting pro-inflammatory gene expression (70). With their known inflammation and atherosclerosis-related functions, collectively these STAT1-dependent integrated genes could serve as new biomarkers and therapeutic targets in human atherosclerosis.

In their meta-analysis study, Mosquera et al. also uncovered a critical role for modulated SMC phenotypes, including contractile SMC, transitional SMC, foam-like SMC and fibromyocytes, in CAD, myocardial infarction, and coronary calcification. They also identified fibromyocyte/fibrochondrogenic SMC markers (LTBP1 and CRTAC1) as proxies of atherosclerosis progression and validated these through omics and spatial imaging analyses (7). In our study, we identified a subset of 511 differentially expressed genes and 117 STAT1-dependent integrated genes, specific for the VSMC population and connected to VSMC function and atherosclerosis. These genes displayed arterial bed-specific and VSMC-subtype dependent expression profiles, being consistent with the description of multiple VSMC phenotypes during atherosclerosis (71). Together, this implies that MØ-dependent and VSMC expressing STAT1-integrative genes consist of different subsets with their unique expression profiles in human atherosclerotic and non-atherosclerotic arteries. Moreover, these different gene subsets reflect cell-type specific functions, connected to inflammation and atherosclerosis on the one hand and more VSMC-related functions on the other.

In order to compare MØ-dependent STAT1-integrative gene expression between human and mouse atherosclerosis, we also analyzed a single-cell RNA-seq data set of aortic lesions from a LDLr-/- HFD mouse models, comparing LFD (Control) *vs* HFD (12 weeks: Late disease). Integrative analysis of the LDLr-/- derived sc-RNAseq data set, revealed dynamic changes in various cell populations, including the MØ sub-types: ISG-expressing immune cells and non-classical monocytes. When comparing late disease *vs* control groups, both ISG-expressing immune cells and non-classical monocytes displayed higher numbers in the lesion group. Moreover, our differential expression analysis identified 400 genes that were differentially expressed in non-lesion vs lesion group in both MØ subtypes. KEGG enrichment analysis highlighted the relation of these genes to immunity and atherosclerosis and functional overlap with differentially expressed genes in MØ sub-types of human atherosclerotic lesions. Among these differentially expressed genes, 73 STAT1-dependent integrative genes could be recognized, with lesion-dependent changes in ISG-expressing immune cells and non-classical monocytes. This was in line with observations presented by Örd and colleagues (8). Using similar data sets, they identified 12 disease-associated cell states that were further characterized by gene set functional profiling, ligand-receptor prediction, and transcription factor inference. Accordingly, three MØ-derived cell states were identified, called Spp1þ MPs, Ccl4þ MPs, and Stmn1þ MPs, which were increased in late disease conditions. In addition, Vcam1þ SMC state genes were identified, which contributed most to SNP-based heritability of CAD (8).

To further substantiate the overlap between human atherosclerosis and mouse atherosclerosis models and the potential involvement of MØ-dependent STAT1-integrative genes we additionally included a bulk RNA-seq data set from HFD fed ApoE knockout mice aorta (48). Based on comparative analysis between atherosclerotic plaques from human patients and ApoE-/- and LDLr-/- high fat diet mouse models, we were able to select 24 STAT1-integrative gene set, specifically expressed in MØ sub-types across human and mouse atherosclerotic lesions (LDLR-/- HFD). Characterization in human atherosclerotic plaques confirmed MØ subtype and arterial bed specific expression of this subset of STAT1-integrative genes. Moreover, protein-protein interaction network analysis predicted functional and physical associations with STAT1 acting as a hub. Also, IFNγ-mediated active transcription of these 24 genes in MØ coincided with prominent promoter STAT1-PU.1 co-binding to GAS and ISRE sites, increased histone methylation and acetylation and chromatin accessibility, a characteristic of MØ-dependent STAT1-integrative genes. Finally, these genes were found to be strongly connected to phenotypic traits such as cardiovascular diseases, cholesterol and lesions, which correlated with a previous proven role in atherosclerosis, including involvement in inflammatory response and chemotaxis, lipid metabolism and homeostasis, apoptosis and cellular stress, extracellular matrix and plaque stability and immune regulation. For example, the CCL2-CCR2 and CCL5-CCR1/CCR5 chemokine axes are critical for monocyte recruitment and early atherogenesis. Blocking these pathways could be a potential therapeutic strategy for atherosclerosis (72, 73). IFITM1 and IFITM3 are two interferon-induced transmembrane proteins that might play significant roles in the pathophysiology of atherosclerosis, particularly through their involvement in inflammation, endothelial function, and vascular health (74). The activation of c-Jun is also linked to the inflammatory processes in atherosclerosis (75). Animal model studies have shown that many cathepsin family genes, including CTSC (Cathepsin C), are highly expressed in atherosclerotic plaques (76). DDIT3 (DNA damage-inducible transcript 3) expression is positively correlated with arterial calcium content and intima-media thickness (IMT) in children with chronic kidney disease (CKD), suggesting it contributes to accelerated arterial calcification and remodeling (77). High temperature requirement A1 (HTRA1) is primarily known for its proteolytic activity, which involves the cleavage of various extracellular matrix components. This activity is crucial for maintaining vascular homeostasis and regulating processes such as angiogenesis and vascular remodeling (78, 79). Id3 plays a protective role against atherosclerosis. Indeed, Id3-/-ApoE-/- mice develop significantly more atherosclerosis compared to Id3+/+ApoE-/- mice, demonstrating a direct relationship between loss of Id3 and increased atherosclerosis (80). Ly6e appears to be a marker of certain MØ subsets that are enriched in progressing atherosclerotic plaques, suggesting it may play a role in disease progression (47). In cardiovascular disease, endothelial polarity proteins like MARCKSL1 help establish endothelial identity and have atheroprotective effects. Endothelial cells secrete extracellular vesicles containing MARCKSL1 in a polarized manner, which can alter monocyte and smooth muscle cell behavior in a compartment-specific way (81). NUPR1 is a critical player in the cellular response to stress and oxidative damage. NUPR1’s activation has been linked to increased cardiovascular risk (82, 83). Likewise, Txn1, or Thioredoxin-1 helps mitigate oxidative stress within the vascular system (84). Recent studies have identified PLAUR (Plasminogen activator, urokinase receptor) as an effective diagnostic marker for atherosclerosis lesion progression. Elevated expression levels of PLAUR have been correlated with the severity of atherosclerosis in both human and mouse models (85). Prdx1 (peroxiredoxin 1) deficiency in MØ leads to increased susceptibility to oxidative stress and impaired clearance of modified LDL due to defective lipophagic flux, thereby promoting atherosclerosis in apoE-deficient mice (86). Elevated levels of Serping1, also known as C1-inhibitor (C1INH) may indicate a negative prognosis for coronary collateral development, which is important for maintaining blood flow in ischemic conditions (87).

The dual role of C1qB in atherosclerosis—both promoting inflammation and providing protective effects—highlights its complexity in disease progression. It has been suggested that the balance between these opposing effects could influence the development and stability of atherosclerotic plaques (88–90). IRF8 appears to play a complex, cell type-specific role in atherosclerosis development, with myeloid IRF8 promoting plaque formation (91, 92). The enzyme cholesterol 25-hydroxylase (CH25H) converts cholesterol into 25-hydroxycholesterol (25-HC), an oxysterol that accumulates in human atherosclerotic lesions, promoting inflammation and plaque instability (93). The interleukin-1 receptor antagonist (IL-1Ra), encoded by the IL1RN gene, acts as an important anti-inflammatory brake on IL-1 signaling in the vasculature (94). SOCS3 (Suppressor of Cytokine Signaling 3) affects macrophage behavior within atherosclerotic plaques. It has been observed that loss of SOCS3 can induce an anti-inflammatory MØ phenotype, which is beneficial in limiting vascular inflammation and atherosclerosis progression (95, 96). Thrombospondin-1 (TSP-1) is known to modulate inflammatory responses within atherosclerotic plaques. Studies indicate that TSP-1 deficiency leads to increased macrophage infiltration and higher levels of inflammatory cytokines in plaque environments. Specifically, in Thbs1-/- mice, a significant increase in MØ-induced inflammation was observed, correlating with accelerated plaque necrosis and degradation of elastic lamina due to matrix metalloproteinases (97, 98). Tnfaip2 (Tumor Necrosis Factor Alpha-Inducible Protein 2) enhances inflammatory responses in atherosclerotic lesions. In particular, Tnfaip2 deficiency has been shown to reduce inflammatory cytokine levels and plaque lesions in mouse models of atherosclerosis, indicating its pro-inflammatory role in disease progression (99). Together, this confirms the important role of MØ-dependent STAT1-driven transcription in atherosclerotic plaque formation and specifically identifies a STAT1-dependent gene signature that could help monitor plaque progression in human atherosclerotic disease. With the low levels of IFNγ detected in the human and mouse artery tissue scRNAseq data sets we cannot rule out that IFNγ may not be the, or not the only, cytokine that induces a STAT1 pattern of gene expression in atherosclerosis lesions in as much that other cytokines can also induce GAF and/or ISGF3 activity in myeloid cells including type I interferons in some settings, IL-6, and IL-27.

The advent of single-cell sequencing technologies has enabled study of gene expression and regulation in disease and development at the single-cell level. For instance, scRNA-seq studies have resolved the cellular diversity and gene signatures in human and murine atherosclerotic lesions (42, 100–103) as well as non-lesion arteries (8, 39). Using data mining of human plaque transcriptomes, we previously unraveled increased expression of STAT1-dependent proatherogenic genes in human atherosclerosis. As such, by comparing publicly available carotid (*n* = 124) and coronary (*n* = 40) artery plaque transcriptomes, we identified a 72 gene “plaque signature” that predominantly consisted of STAT1-target genes (21). In addition, we recently identified the novel multi-IRF inhibitor, ALEKSIN, which exhibited genome-wide inhibition potential toward IRF-, STAT-, and NF-κB-mediated transcription, similar to the known multi-STAT inhibitor STATTIC. Furthermore, we discovered a signature of 46 ALEKSIN and STATTIC commonly inhibited pro-atherogenic target genes, predominantly linked to MØ subtypes present in aortic plaques in HFD fed LDLR-KO mice (48). Based on our recent and current findings and in analogy to biomarker assays connected to cancer and transplant rejection (4, 21), a predefined STAT1-target gene signature could be developed as a novel diagnostic tool to monitor and diagnose plaque phenotype in human atherosclerosis. The incorporation of MØ-specific STAT1-target genes in this gene signature would be highly valuable as it potentially allows monitoring plaque-specific inflammatory responses in a cell-type dependent manner. Together with our recently developed STAT and IRF inhibition strategies during vascular inflammation (4, 48, 104), this may open a promising avenue towards development of targeting and monitoring therapies in the treatment of atherosclerosis.

We acknowledge some limitations in our study my arise from the sourced datasets included in this meta-analysis. First, while the integration of multi-omics data in bone marrow-derived MØ provides a comprehensive view of the transcriptional landscape, it may not fully capture the temporal dynamics of TF binding and epigenetic modifications over the course of atherosclerosis progression. The use of only short-term time points in the analysis may overlook critical changes that occur at other stages of disease development. Second, in the human atherosclerosis sc-RNAseq analysis part, non-lesion samples were derived from patients with non-ischemic dilated cardiomyopathies, and inflammatory cell populations could be consequences of myocardial inflammation or secondary subclinical diffuse intimal thickening. Third, in the human as well as the mouse atherosclerosis sc-RNAseq analysis part, while the majority of the cell types were balanced across samples, it is difficult to separate biologically meaningful processes or technical factors. Fourth, we acknowledge the need for systematic protein-level and experimental validation of our preselected 24 STAT1-integrative gene set to confirm their precise functions in atherosclerosis. Fifth, the study primarily focuses on the role of STAT1 in MØ, potentially underestimating the contributions of other transcription factors, cell types and signaling pathways involved in atherosclerosis. Sixth, with the low levels of IFNγ detected in the human and mouse artery tissue scRNAseq data sets we cannot rule out that IFNγ may not be the, or not the only, cytokine that induces a STAT1 pattern of gene expression in atherosclerosis lesions. Future studies should aim to address these limitations by incorporating longitudinal analyses and exploring the interactions between various cell types within the atherosclerotic microenvironment.

## Materials and methods

### MØ isolation and differentiation

Bone marrow-derived MØ (BMDM) samples from C57BL/6 mice of either sex were isolated from femur and tibia by flushing the bones followed by red blood cell lysis with ACK buffer and centrifugation at 1500 RPM. Cells were differentiated for 9–10 days in Dulbecco’s modified Eagle’s medium (DMEM) (PAS Wrocław) supplemented with 15% fetal bovine serum (FBS, Thermo Fisher Scientific), 100 units/ml Penicillin and 100 units/ml Streptomycin (Pen/Strep) (Sigma-Aldrich) and M-CSF (PeproTech) on 6-well culture-dishes. Cells were cultured at 37°C and 5% CO2. BMDMs were stimulated with IFNγ (10 ng/ml, TFS) at specified time points (0, 0.5, 2, 4, 8, 24h).

### RNA isolation and RNA-seq library preparation

Total RNA was isolated using TRI-REAGENT (MRC) followed by a column-based Total RNA Zol-Out™ D kit (A&A Biotechnology) based on manufacturer’s protocol. RNA was quantified using Qubit RNA HS (High Sense) assay kit (TFS) and quality was assessed using Agilent RNA 6000 Nano Reagents kit (Agilent Technologies) according to the manufacturer’s protocol. Only RNA with RNA Integrity Number (RIN) > 9 was considered for library preparation. RNA-seq libraries were prepared in three biological replicates from 1ug of total RNA using NEBNext^®^ Ultra™ II RNA Library Prep Kit for Illumina^®^ (NEB) together with NEBNext Poly(A) mRNA Magnetic Isolation Module (NEB) and NEBNext^®^ Multiplex Oligos for Illumina^®^ (NEB) according to manufacturer’s protocol. Libraries were quantified using Qubit dsDNA HS assay kit (TFS) and quality and fragment distribution were examined with Agilent High Sensitivity DNA kit (Agilent Technologies). Sequencing was performed on the HiSeq X (150PE) by Macrogen Europe B.V.

### ApoE KO-based atherosclerosis mouse model

The ApoE KO HFD model was essentially performed as described previously (65). The experiment was conducted on 16 ten-week-old house mice (Mus musculus) B6.129P2-ApoEtm1Unc/J (purchased from Jacksons Laboratory). Breeding and animal experiments were performed in the animal facility of the Wielkopolskie Centrum Zaawansowanych Technologii (WCZT) in Poznań. All mice work was performed in accordance with the agreement of the Poznan Local Ethical Committee under approval number 16/2019 and 42/2021. Animals were divided into two groups (2x n=8) with mixed sexes. The first group was fed a standard low-fat chow diet (LFD) and the second group of mice was fed a high-fat diet (HFD; High Fat, +7.5 g/kg Cholesterol, Experimental diet, 10.7% fat, Ssniff S GmbH). After a week of acclimatization and handling, 8-week-old ApoE KO mice were subjected to LFD or HFD for 12 weeks, during which HFD fed mice developed atherosclerotic deposits (48).

For RNA isolation, frozen tissues were transferred into Trizol (A&A Biotechnology) and homogenized using a manual Omni tissue homogenizer and dedicated hard tips. All the following steps of RNA isolation were carried out according to Total RNA Zol-Out (A&A Biotechnology) protocol for the rapid purification of ultra-pure total RNA. RNA-seq library preparation followed the same procedure as for macrophages (see above).

### RNA-seq data analysis

The quality of sequencing reads, and potential adapter contaminations were evaluated by FastQC (0.12.1) (http://www.bioinformatics.babraham.ac.uk/projects/fastqc/). Low-quality sequences with a Phred score of < 20 were removed by Trim_Galore(0.6.10) (105). Afterward, the filtered reads were aligned to the mouse genome (GRCm38/mm10) with a fast and efficient spliced aligner tool STAR (2.7.10) (106). FeatureCounts (1.6.2) was employed for the summarization of mapped reads into genomic attributes (107). Genes with counts lower than 10 at any time points were filtered out. To determine differentially expressed genes (DEG), DESeq2 (1.40.2) package (108) in R (4.3.3) was used. The likelihood ratio test (LRT) was implemented to identify genes that respond to IFN treatment over time. False discovery rate (FDR)-adjusted q-values (5% threshold) were calculated by Benjamini–Hochberg procedure. The log2(fold change) FC also was calculated for each gene. Genes with adjusted p-values (padj) less than 0.05 and |log2FC| > 1 were considered as DEGs.

### ATAC-seq data processing

To identify open chromatin regions, the raw sequencing ATAC-seq data was analyzed using nfcore/atacseq pipeline (2.1.2) (109). This pipeline is a robust and reproducible method for the processing of ATAC-seq data, which is based on Nextflow (23.04.1). The nfcore/atacseq pipeline includes several stages. Generally, reads were aligned to the mouse genome (GRCm38/mm10) using bwa aligner (0.7.17-r1188) (110), followed by peak calling by MACS2 (111).

### ChIP-seq data analysis

The raw sequencing ChIP-seq data was analyzed using ENCODE Transcription Factor and Histone ChIP-Seq processing pipeline (https://github.com/ENCODE-DCC/chip-seq-pipeline2) with default parameters as recommended by ENCODE Consortium (112). Briefly, the sequencing reads were aligned to the mouse genome (GRCm38/mm10) using bowtie2 (2.3.4.3) (113). Then, duplicates were marked using Picard Tools (2.20.7)(https://github.com/broadinstitute/picard). Peak calling for transcription factors and histones was performed using SPP and MACS2, respectively with FDR threshold set to 0.01. Afterwards, Irreproducible Discovery Rate (IDR) was implemented to identify an optimal number of reproducible peaks between biological replicates, with an IDR score threshold of 0.05.

### Correlation analysis

The normalized peak files related to ATAC-seq and ChIP-seq data were merged using merge function in bedtools package (114), followed by counting peaks in each datasets using featureCounts (107) and combining all the count tables into single table for the assessment of correlation using Pearson method.

### Identification of differential peaks and integration of datasets

To standardize all sequence alignments from different datasets (ATAC-seq, STAT1, PU.1, H3K27ac, H3K4me1 and H3K4me3), “Tag Directory” was created using the Homer function makeTagDirectory (50). Then, to find peaks that are differentially enriched between two conditions, the Homer function getDifferentialPeaks was implemented. These normalized differental peaks located in the promoter region region (-3000, 3000 bp from TSS) were further selected by ChIPseeker (1.36.0) (115), followed by preparing a list of mutual peaks associated with above-mentioned datasets. Next, those peaks were integrated with up-regulated, adjusted p-value (padj < 0.05) genes from our in-house RNA-seq data using BETA tool (1.0.7) (116). The upregulated direct target list was selected as integrative genes for downstream analysis.

### Identification of TF binding motifs and distribution of epigenetic marks near the promoter regions

To quantify TF binding of GAS, ISRE & PU.1 motifs in the promoter regions, the Homer function annotatePeaks.pl was implemented on PU.1 and STAT1 peaks in the IFNγ-treated group using GAS & ISRE motifs from our previous motif analysis (117) and PU.1 motifs from Homer Motif Library (http://homer.ucsd.edu/homer/custom.motifs). To measure the distibution pattern of acetylation and methylation marks and also PU.1 and STAT1 binding sites, Homer function annotatePeaks.pl was employed on the peaks related to integrative genes.

### Single-cell RNA-seq data analysis

Raw count matrices from each library across the four different studies (mouse and human) were downloaded from GEO and Zenodo (**Supplementary Table S1**). The library processing was performed based on the workflow suggested by Mosquera et al. (7). Briefly, 17 libraries were processed using Seurat (4.3.0) (118) running in R version 4.3.3. To remove the doublets and ambient RNA, scDblFinder(1.16.0) (119) and Celda::DecontX(1.18.1) (120) R packages were employed, respectively. Then, the decontaminated raw count matrices were further filtered to keep the cells that follow 1) >=200 and <=4000 uniquely expressed genes 2) >=200 and <= 20000 UMIs 3) <=10% of reads mapped to the mitochondrial genome 4) <= 5% of reads mapped to hemoglobin genes. Filtered count matrices were normalized using SCTransform (121). During SCTransform normalization, parameters vst.flavor = “v2” and vars.to.regress = c(“S.Score”,”G2M.Score”) were implemented to take into account for sequencing depth variability and cell cycle variance, respectively. Then, dimensionality reduction of the normalized counts matrix was implemented using Principal Component Analysis (PCA), follwed by applying Uniform Manifold Approximation and Projection (UMAP) non-linear dimensionality reduction using the first 30 PCs.

To intergrate scRNA libraries and remove batch effects, a list of species-specific processed Seurat objects was created, followed by the extraction of 3000 highly variable genes across datasets using SelectIntegrationFeatures. Next, PCA was run across each library using the 3000 variable genes, followed by identification of integration anchors using dimensional reduction method “Reciprocal PCA (rPCA)”, which is an efficient method with respect to the running time and conservation of biological signal. Due to smaller number of mouse datasets, canonical correlation analysis (CCA) were employed instead of rPCA method. The batch-corrected count matrix was then used for PCA dimensionality reduction, creation of the shared-nearest-neighbors (SNN) graph using 30 PCs, and Louvain clustering followed by visualization with UMAP embeddings.

To annotate cell types in a robust manner, we used a blend of automated and manual approaches. For human datasets, we first annotated the integrated data using human cell atlas “Tabula Sapiens (TS)” with a specific focus of Immune and vasculature subset of this atlas (122). To be consistent with our SCTransformed-integrated datasets, immune and vasculature TS subsets were re-normalized using SCTransform prior to Seurat’s label transfer. We also took advantage of the curated lists of gene markers related to immune and mural cell types in human (7) and in mouse (8) to assess the enrichment score of these genes in our integrated datasets using UCell R pacakge (2.6.2) (123). Additionally, we also obtained gene markers for each of the SNN-derived clusters using the PrepSCTMarkers and FindAllMarkers functions from Seurat (v4.3.0). Moreover, ScType was implemented for fully-automated cell-type identification based on their comprehensive cell marker database as background information (44). Altogether, the cell-annotations of our integrated datasets were finalized using TS atlas, UCell enrichment scores, gene-specific markers for each cluster, ScType predictions and manual confirmation.

### Pseudo-bulk single-cell RNA-seq analysis

For differential expression analyses, we initially identified the 3000 most variable genes, followed by retaining these variable genes to accelarate weights computation. We then implemented Zero-Inflated-based Negative Binomial Wanted Variation Extraction (ZINB-WaVE) approach (124) using R zinbwave package (1.24.0) to identify excess zero counts and generate gene- and cell-specific weights. The weights were computed taking into account sex, arterial bed and condition as covariates, where applicable. Next, DESeq2 method (108) was applied on ZINB-adjusted expression data using single-cell data suitable likelihood ratio test.

### Transcription factor activity inference

To infer TF activity, we focused on a specifc list of curated TFs including STAT and IRF familes in DoRothEA R package (1.12.0) (125). TFs with high confidience scores were selected and TF activities were then estimated with the R package VIPER (1.36.0) (126) using the filtered list of regulons and processed Seurat objects which were constructed based on either all the genes or integrative genes. We calculated mean TF activities accros different human macrophage subtypes for either of these seurat objects.

## Data Availability

The datasets presented in this study can be found in online repositories. The names of the repository/repositories and accession number(s) can be found below: https://www.ncbi.nlm.nih.gov/, GSE276418 and GSE270260. The custom scripts and PU.1, GAS and ISRE motifs used in this publication are available on GitHub https://github.com/peculiar97/Atherosclerosis_Multiomics_scRNAseq.

## Supplementary material

Supplementary Figure 1 Vascular smooth muscle cell population in human atherosclerotic plaques. **(A)** The expression profile of differentially expressed genes (lesion *vs* non-lesion) in vascular smooth muscle cell population. The hierarchical cluster analysis generated three distinct clusters. The STAT1-target genes were shown on the left side. **(B)** KEGG pathway analysis of each cluster revealed cluster-specific signaling pathways related to muscle activity. The intersection of each Venn diagram shows the number of integrative genes (STAT1-target genes) in each cluster (Cluster 1, Cluster 2, and Cluster 3).

Supplementary Figure 2 The feature plots derived from single-cell RNA sequencing (scRNA-seq) datasets, illustrating the expression levels of interferon-gamma (IFNγ) in natural killer (NK) and T cell (NK/T) populations for human (left panel) and mouse (right panel) samples. Cells with no detectable IFNγ expression appear as grey.

Supplementary Table S1 Public datasets used in this study and sample metadata.

Supplementary Table S2 Differentially expressed genes (DEGs) in IFNγ-treated Macrophages at specified time points (0, 0.5, 2, 4, 8, 24 hours).

Supplementary Table S3 STAT1-dependent integrative gene list.

Supplementary Table S4 Cell type markers were used for the cell annotation of human atherosclerotic plaques.

Supplementary Table S5 The differentially expressed genes in each human macrophage subtype.

Supplementary Table S6 The differentially expressed genes from high-fat diet (HFD) fed ApoE knockout mice.
