# Supplementary figures and images for: Integrative multi-omics analysis of IFNγ-induced macrophages and atherosclerotic plaques reveals macrophage-dependent STAT1-driven transcription in atherosclerosis
Source: Front Immunol. 2025 Jun 18;16:1590953. doi: 10.3389/fimmu.2025.1590953 (PMC12213720; doi:10.3389/fimmu.2025.1590953)

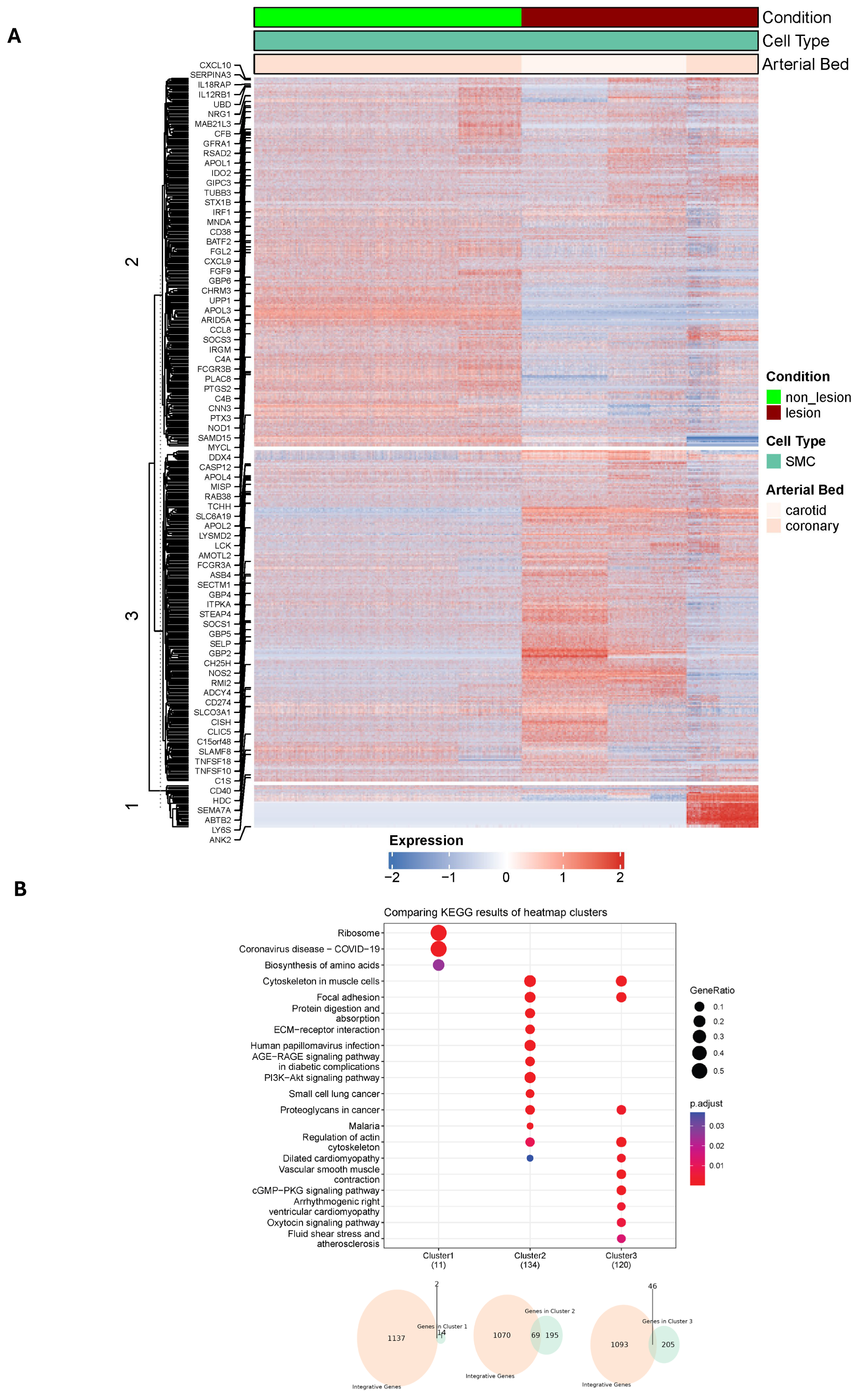

Supplement: Supplementary Figure 1 — Vascular smooth muscle cell population in human atherosclerotic plaques. (A) The expression profile of differentially expressed genes (lesion vs non-lesion) in vascular smooth muscle cell population. The hierarchical cluster analysis generated three distinct clusters. The STAT1-target genes were shown on the left side. (B) KEGG pathway analysis of each cluster revealed cluster-specific signaling pathways related to muscle activity. The intersection of each Venn diagram shows the number of integrative genes (STAT1-target genes) in each cluster (Cluster 1, Cluster 2, and Cluster 3). [file Image1.tif]

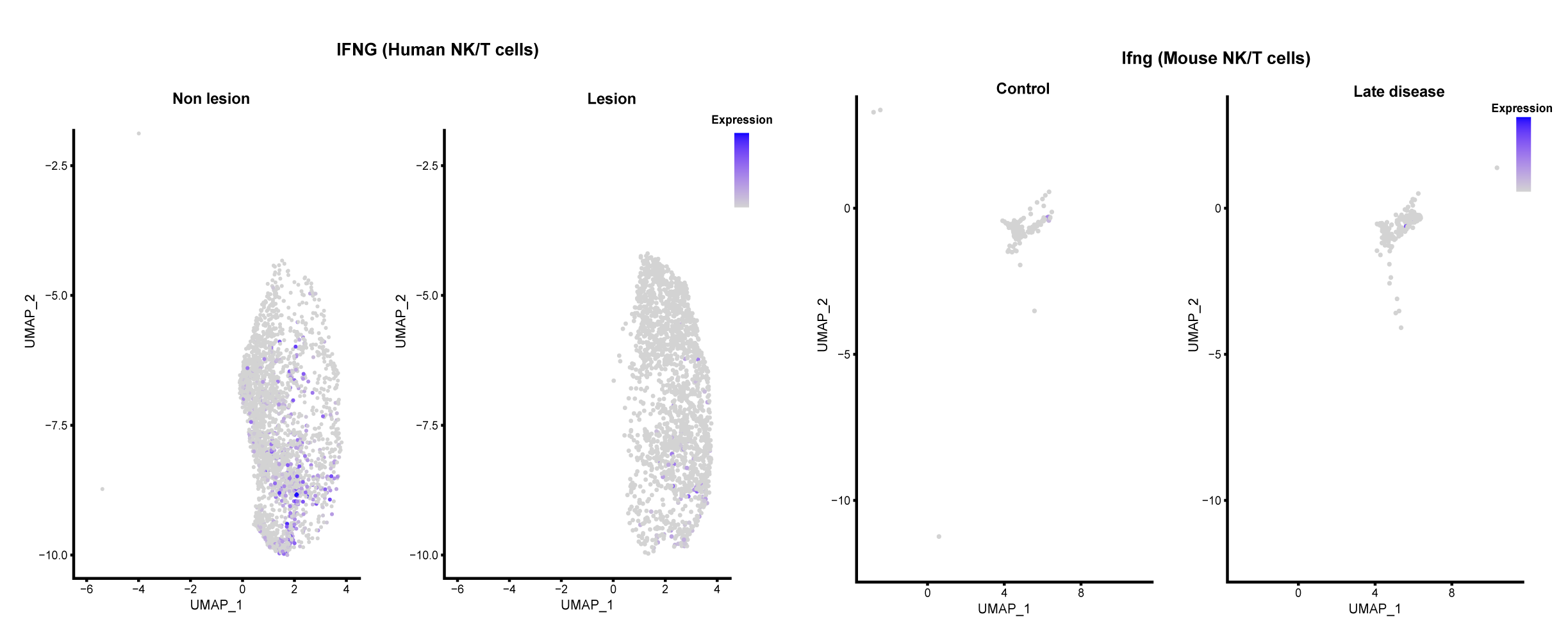

Supplement: Supplementary Figure 2 — The feature plots derived from single-cell RNA sequencing (scRNA-seq) datasets, illustrating the expression levels of interferon-gamma (IFNγ) in natural killer (NK) and T cell (NK/T) populations for human (left panel) and mouse (right panel) samples. Cells with no detectable IFNγ expression appear as grey. [file Image2.tif]
